# Conserved Gene Microsynteny Unveils Functional Interaction Between Protein Disulfide Isomerase and Rho Guanine-Dissociation Inhibitor Families

**DOI:** 10.1038/s41598-017-16947-5

**Published:** 2017-12-08

**Authors:** Ana I. S. Moretti, Jessyca C. Pavanelli, Patrícia Nolasco, Matthias S. Leisegang, Leonardo Y. Tanaka, Carolina G. Fernandes, João Wosniak, Daniela Kajihara, Matheus H. Dias, Denise C. Fernandes, Hanjoong Jo, Ngoc-Vinh Tran, Ingo Ebersberger, Ralf P. Brandes, Diego Bonatto, Francisco R. M. Laurindo

**Affiliations:** 1 Vascular Biology Laboratory, Heart Institute (Incor), University of São Paulo School of Medicine, São Paulo, Brazil; 2 The Wallace H. Coulter Department of Biomedical Engineering, Georgia Institute of Technology and Emory University, Atlanta, USA; 3 Special Laboratory for Cell Cycle, Center of Toxins, Immune-Response and Cell Signaling - CeTICS-Cepid, Butantan Institute, São Paulo, Brazil; 4 Institut für Kardiovaskuläre Physiologie, Goethe University, Frankfurt, Germany; 5 Applied Bioinformatics Group, Institute of Cell Biology & Neuroscience, Goethe University, Frankfurt, Germany; 6 Senckenberg Biodiversity and Climate Research Center (BiK-F), Frankfurt, Germany; 7 Department of Molecular Biology and Biotechnology, Federal University of Rio Grande do Sul, Porto Alegre, Brazil

## Abstract

Protein disulfide isomerases (PDIs) support endoplasmic reticulum redox protein folding and cell-surface thiol-redox control of thrombosis and vascular remodeling. The family prototype PDIA1 regulates NADPH oxidase signaling and cytoskeleton organization, however the related underlying mechanisms are unclear. Here we show that genes encoding human PDIA1 and its two paralogs PDIA8 and PDIA2 are each flanked by genes encoding Rho guanine-dissociation inhibitors (GDI), known regulators of RhoGTPases/cytoskeleton. Evolutionary histories of these three microsyntenic regions reveal their emergence by two successive duplication events of a primordial gene pair in the last common vertebrate ancestor. The arrangement, however, is substantially older, detectable in echinoderms, nematodes, and cnidarians. Thus, PDI/RhoGDI pairing in the same transcription orientation emerged early in animal evolution and has been largely maintained. PDI/RhoGDI pairs are embedded into conserved genomic regions displaying common cis-regulatory elements. Analysis of gene expression datasets supports evidence for PDI/RhoGDI coexpression in developmental/inflammatory contexts. PDIA1/RhoGDIα were co-induced in endothelial cells upon CRISP-R-promoted transcription activation of each pair component, and also in mouse arterial intima during flow-induced remodeling. We provide evidence for physical interaction between both proteins. These data support strong functional links between PDI and RhoGDI families, which likely maintained PDI/RhoGDI microsynteny along > 800-million years of evolution.

## Introduction

Protein disulfide isomerases are redox chaperones from the thioredoxin superfamily, with crucial functions in redox protein folding at the endoplasmic reticulum (ER). The family prototype PDIA1 exhibits dithiol *a* domains and substrate-binding *b* domains, arranged as *a-b-b*′*-a*′. PDI gene family comprises >20 paralogs displaying distinct arrangements of *a* and *b* domains, while a few members such as PDIA8 (Erp27) harbor only redox-inactive *b* domains. Although most PDIs exhibit C-terminal ER retrieval sequences, many are also found at the cell-surface and extracellularly. At these locations, PDIA1 and some other PDIs redox-regulate processes such as cell adhesion, protease shedding, thrombosis, platelet activation^1–7^, vascular remodeling and mechanoadaptation^8^. Also, at yet unclear intracellular locations, at least PDIA1 exerts critical roles in the regulation of Nox family NADPH oxidases, being required for agonist-mediated activation of Nox1 in vascular cells^9,10^ and Nox2 in phagocytes^11,12^. Accordingly, PDIA1 silencing impairs Nox1-dependent vascular smooth muscle cell (VSMC) migration and disorganizes cytoskeletal architecture^10^. This, as well as additional evidences^13^, including a direct binding of PDIA1 to beta-actin^14^, support a role for PDIA1 in cytoskeletal regulation. However, the precise mechanisms whereby PDIA1 interplays with the cytoskeleton remain unclear. PDIA1 silencing promotes impaired activation of RhoGTPases Rac1 and RhoA, which are master molecular switches well-known to control cytoskeletal remodeling^10^. This suggests that RhoGTPases and/or their regulators might be involved in PDI-related cytoskeletal effects. Indeed, analysis of in silico protein-protein interaction networks and experiments of co-immunoprecipitation indicate that PDIA1 can associate with Rho guanine dissociation inhibitor-α (RhoGDIα) in VSMC^10^. RhoGDIs, which comprise 3 known members in humans (α, β and γ), are cytosolic proteins exerting critical roles in RhoGTPase regulation. RhoGDIs act as chaperones stabilizing RhoGTPases^15^ and switching them bidirectionally between cytosol and membrane. Through these actions, RhoGDIs organize RhoGTPase cycling and focal activation. The nature of PDIA1 and RhoGDIα interaction is unknown, and their association is not obvious, given their distinct canonical subcellular locations and functions. Thus, further investigating their cooperation at several hierarchic levels is important to establish plausible mechanistic links between PDIA1 and cytoskeleton/RhoGTPase regulation.

Direct evidence for the co-regulation of two genes can be obtained from their control by the same cis-regulatory elements. While spatial proximity of two genes on the chromosomal organization is a necessary prerequisite for the sharing of cis-regulatory elements, it typically does not suffice as an indication. If, however, local gene order (a phenomenon designated as synteny) is maintained over evolutionary timescales, this points to the possibility that functional constraints have prevented the rearrangement or loss of the involved genes.  Hallmark examples of syntenic and co-regulated genes are the HOX developmental gene clusters, in which the gene order remained virtually unchanged across animal evolution and the corresponding regions in the contemporary animal genomes remained shared syntenic^16^. Microsynteny can be defined as a fine-scale syntenic linkage with no or very few genes interposed between the two syntenic loci^17^. Therefore, the analysis of shared microsyntenic regions in distantly related species, which provide evidence that gene order remained conserved along evolution, provides an indication that the corresponding genes are coregulated and/or interact functionally^17–19^. In this study, we explore the organization pattern, evolutionary conservation and functional implications of the spatial clustering of members of the PDI and RhoGDI family. Our results reveal the existence of three microsyntenic clusters in the human genome, each harboring a member of the PDI family next to a member of the RhoGDI family. We provide evidence that the presence of these three clusters is widely conserved in vertebrates, and that they arose by two subsequent cluster duplication events in the last common ancestor of vertebrates. However, the arrangement of a primordial RhoGDI next to a PDI gene appears substantially older and is detected in nematodes and in cnidarians. Since PDI/RhoGDI clustering is not found in sponges, ctenophores or in the unicellular closest relatives to the animals, we tentatively date the cluster emergence to the last common ancestor the bilateria share with the cnidarians. Further investigation revealed that the evolutionary maintenance of the PDI/RhoGDI gene microsynteny correlates with their functional cooperation, documented by evidences of coregulation, coexpression and physical interaction of their protein products. Such functional interactions may have constrained the disruption of their syntenic locations along evolution. These data further support strong mechanistic links between PDI and RhoGDI/RhoGTPase families potentially affecting cytoskeletal organization.

## Results

### Characterization and evolutionary history of PDI/RhoGDI synteny

#### Phylogenetic analysis of PDI/RhoGDI families and characterization of their microsynteny in humans  

The PDI gene family is represented by >20 paralogs in humans. We have identified orthologs to 8 of these genes in a selection of taxa spanning the eukaryotic tree of life and reconstructed the evolutionary history of this gene family using maximum likelihood (Supplementary Fig. S1). This reveals that PDI diversification already started in the last common ancestor of the eukaryotes (LECA, Supplementary Fig. S1). Thus, PDIs represent an evolutionarily old gene family that appears to have evolved from domain duplications dating back to an ancestral single thioredoxin-domain prokaryote precursor^20,21^. One of these evolutionarily old PDI lineages gave rise to three of the human PDIs: PDIA1 (P4HB), PDIA2 (PDIp) and PDIA8 (Erp27). These three paralogs exhibit a substantially close relation and emerged by two subsequent gene duplications in the last common ancestor of the vertebrates. For the remainder of the manuscript, we refer to this sub-family as the PDIA1 gene family. We subsequently reconstructed the evolutionary history of the RhoGDI gene family, and a picture strikingly similar to that of PDIA1 family emerged (Supplementary Figure 2). Resembling the evolutionary history of the PDIA1 gene family, the RhoGDIs can also be traced back to LECA, and diversified into its three paralogous copies α, β and γ in the last common ancestor of the vertebrates. Analysis of evolutionary trees using distance-based methods (neighbor joining) reached essentially the same conclusions (data not shown).

Remarkably, the similar evolutionary trajectories of the PDIA1 gene family and the RhoGDI gene family coincide with a tight physical linkage of their respective members in the human genome. For each RhoGDI paralog, we detected a flanking member of the PDIA1 gene family, as follows: RhoGDIα is placed next to PDIA1 (P4HB gene) on chromosome 17, with 7.1 kb intergenic distance; RhoGDIβ is placed next to PDIA8 (Erp27) on chromosome 12, with 2.9 kb intergenic distance; RhoGDIγ is placed next to PDIA2 (PDIP) on chromosome 16, with 0.14 kb intergenic distance (Table 1). In all three cases, the relative transcription direction of the two genes is the same, providing initial evidence that the three clusters emerged by two subsequent en block duplications of a primordial gene pair. While all RhoGDI genes are microsyntenic to a specific PDI gene, extensive analysis of several other PDI family genes, as well as genes related to RhoGTPase family and their regulators did not reveal proximity, respectively, to RhoGTPase-related or thioredoxin superfamily genes (not shown).

**Table 1:** Gene location and main tissue expression of PDI and RhoGDI pairs addressed in the present study.

Genes	Locus	Gene position	Distance between genes	Sense reading	Tissue expression
PDIA1(P4HB) AHRGDIα	17q25.3	81,843,159–81,860,694 81,867,721–81,871,406	7.1 Kb	Reverse	Ubiquitous
PDIA2 AHRGDIγ	16p13.3	283,152–287,215 268,727–283,010	0.14 Kb	Forward	Brain, pancreas, lung, kidney and testis
PDIA8 (Erp27) AHRGDIβ	12p12.3	14,914,035–14,939,082 14,942,017–14,961,72	2.9 Kb	Reverse	Hematopoietic cells

#### Evolutionary age and history of the PDI/RhoGDI synteny

**Figure 1:**
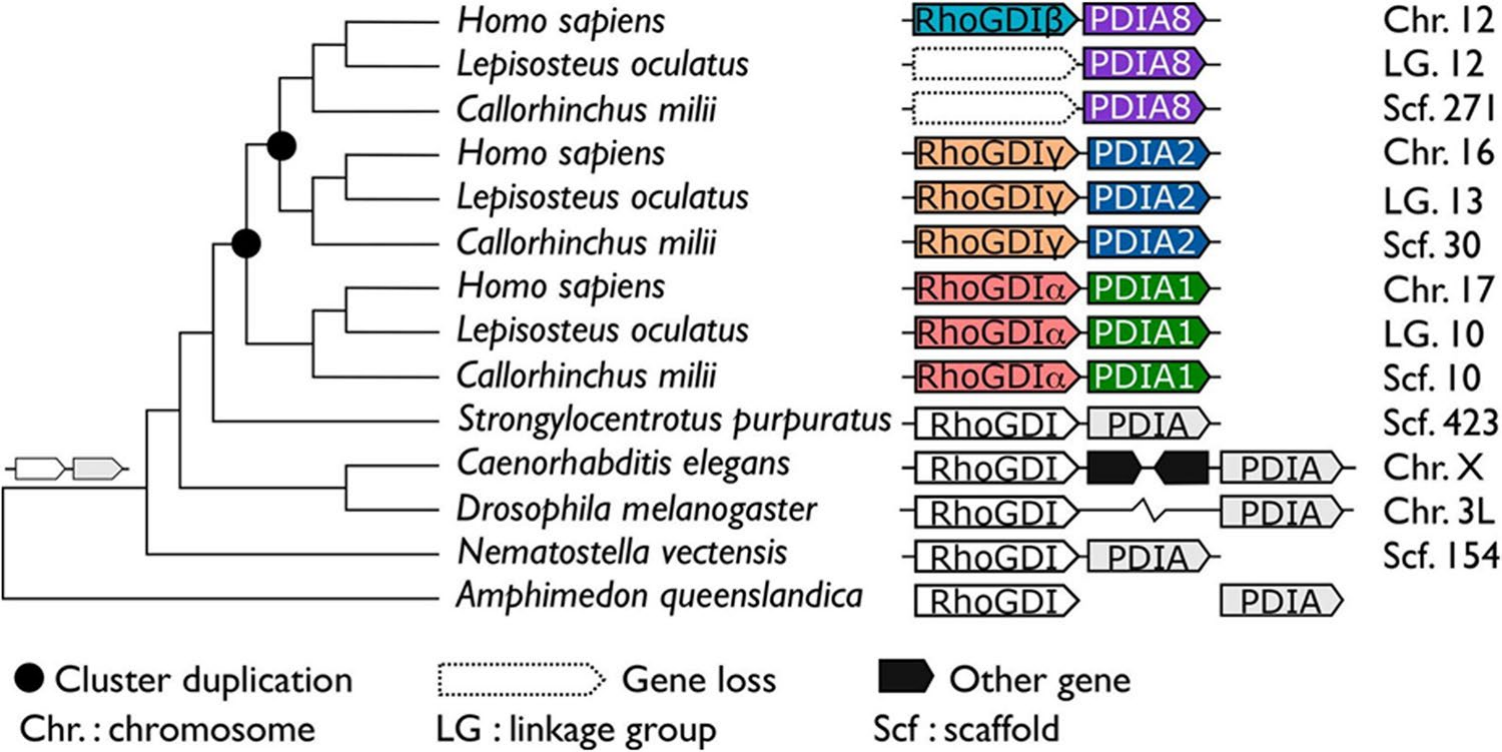
Evolutionary trajectory of the PDI/RhoGDI gene cluster across the animal phylogeny. The emergence of the gene cluster dates back to the last common ancestor shared between bilateria and the cnidarians (*N*. *vectensis*). In earlier branching animal, e.g. sponges (*A*. *queenslandica*) or ctenophores (not shown), orthologs to the participating genes are unlinked. In the arthropods, represented by *D*. *melanogaster*, the cluster has been disrupted. Two subsequent rounds of cluster duplications in the last common ancestor of the vertebrates gave rise to the three contemporary paralogous gene clusters. The tree topology follows the accepted animal phylogeny, the vertebrate subtrees are arranged according to Supplementary Figure 1 (maximum likelihood PDI family tree). *H*. *sapiens* – Sarcopterygii; *L*. *oculatus* – Actinopterygii; *C*. *milii* – Chondrichthyes; *S*. *purpuratus* – Echinodermata; *C*. *elegans* – Nematoda; *D*. *melanogaster* – Arthropoda; *N*. *vectensis* – Cnidaria; *A*. *queenslandica* – Porifera. The genomic localizations in the individual species are given next to the cluster representation. In *D*. *melanogaster*, the two genes reside on the same chromosome, yet at a distance of >1 Mb.

**Figure 2:**
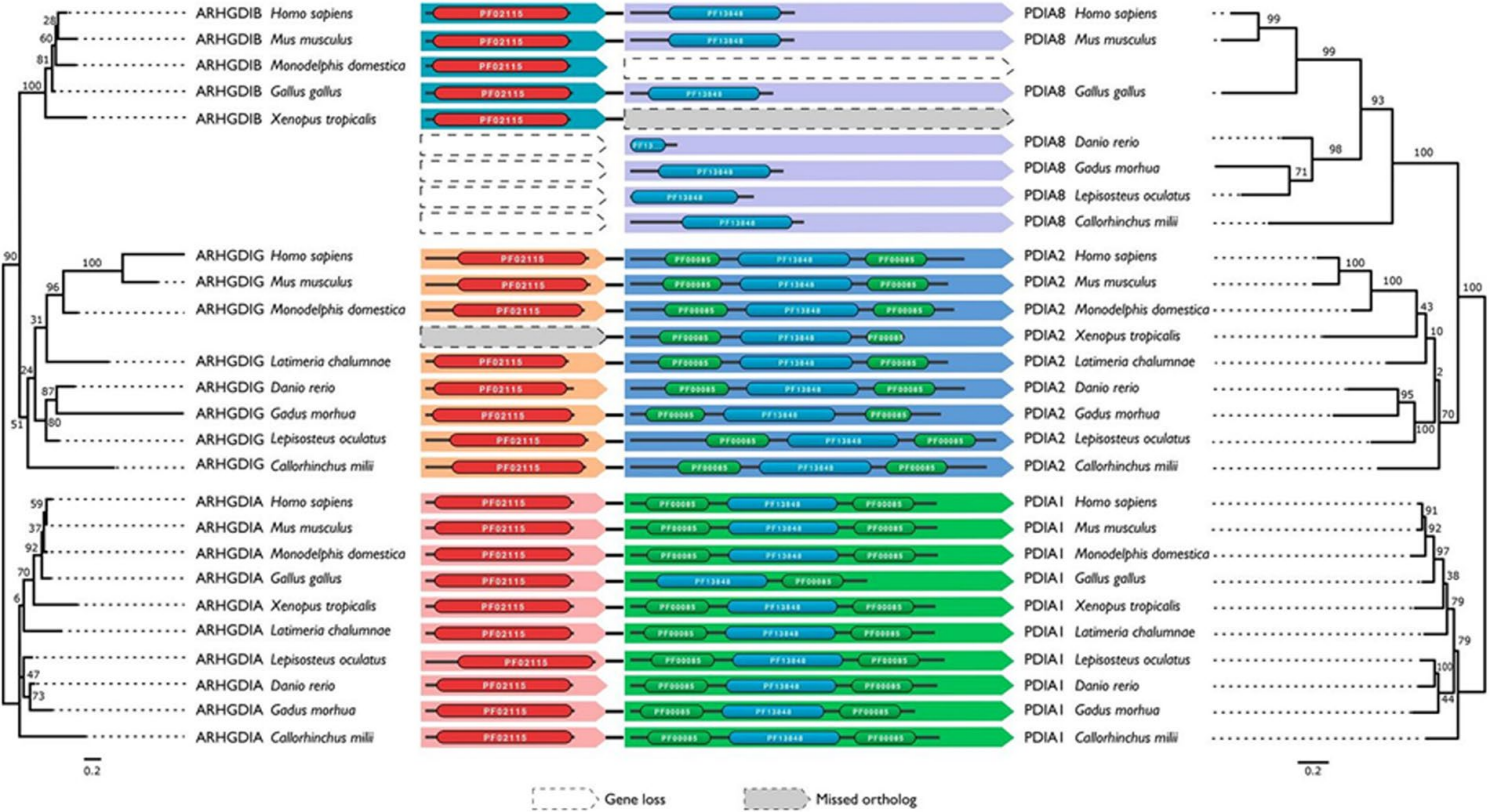
Evolutionary histories of the PDI and RhoGDI paralogs, respectively, in the three human PDI/RhoGDI gene clusters. The trees are based on the maximum likelihood phylogeny of the PDIs and RhoGDIs, respectively, but were adapted for congruency with the species phylogeny. Topology testing revealed that the adapted topology is not significantly worse compared to the ML topology (SH test: p > 0.05). Branch labels denote percent bootstrap support. The Pfam^83^ domain architectures of the individual PDI and RhoGDI proteins are shown next to the leaf labels: PF02115 – Rho_GDI; PF13848 – Thioredoxin_6; PF00085 – Thioredoxin. The architectures are connected if the genes reside next to each other in the genome of the respective species. The orthologous groups, PDIA1, PDIA2, PDIA8 and correspondingly, RhoGDIα, RhoGDIγ and RhoGDIβ are indicated by the same background color. In two cases, an ortholog was not predicted, while gene order indicates its presence (‘missed ortholog’).

**Figure 3:**
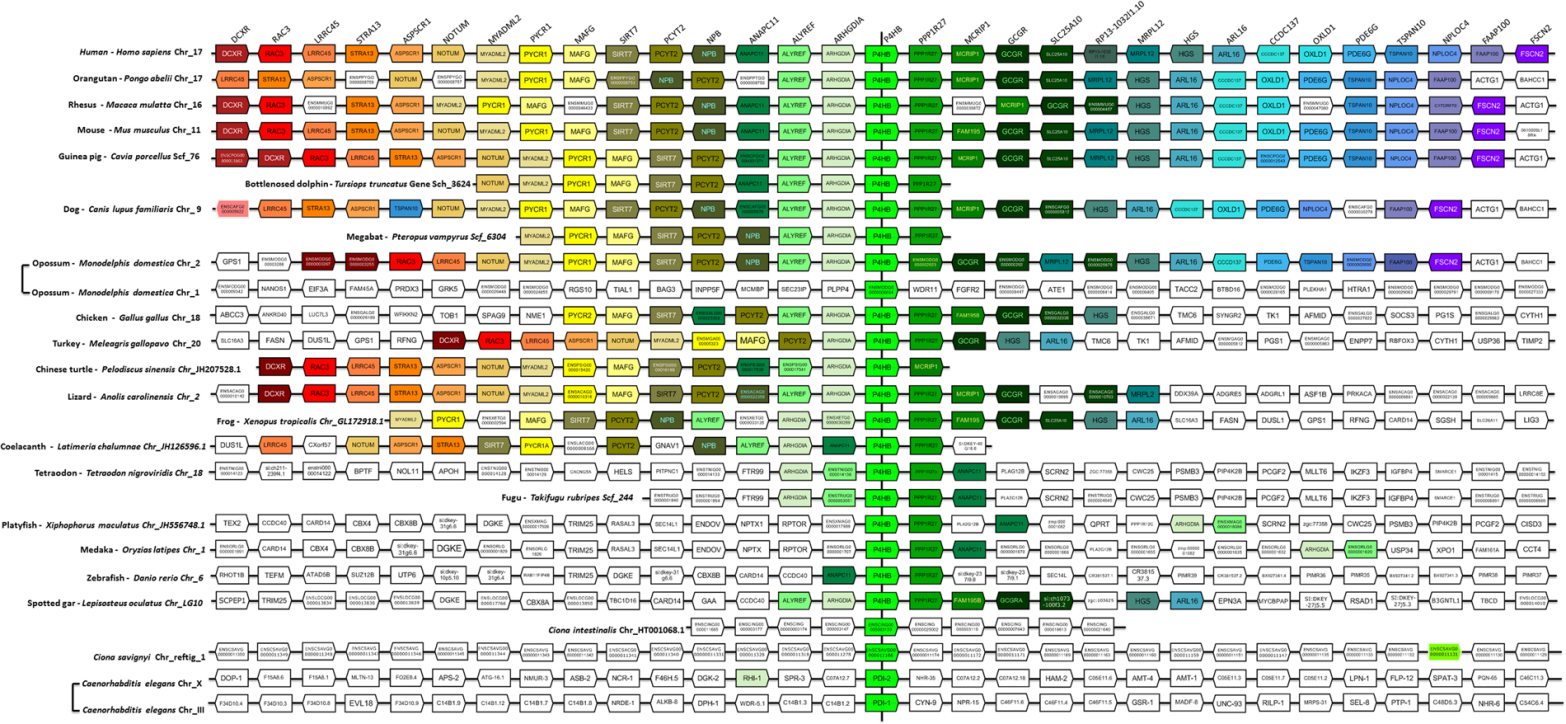
Alignment of the genomic region containing PDIA1/RhoGDIα cluster in Eukaryota. Gene names and positioning are based upon the Genomicus database (see methods). All species are aligned against *Homo sapiens*. The map is centralized in PDIA1 (P4HB)/RhoGDIα (ARHGDIA) gene pair. Genes are aligned in columms and kept in the order in which they appear in chromosomes (Chr) and scaffolds (Scf), without consideration for distance, while the transcriptional sense is represented by the pentagon tip. All orthologs are drawn with the same color and the lettering on the top refers to the *Homo sapiens* genes. In addition to the main genes of interest, the neighboring genes are included for reference.

**Figure 4:**
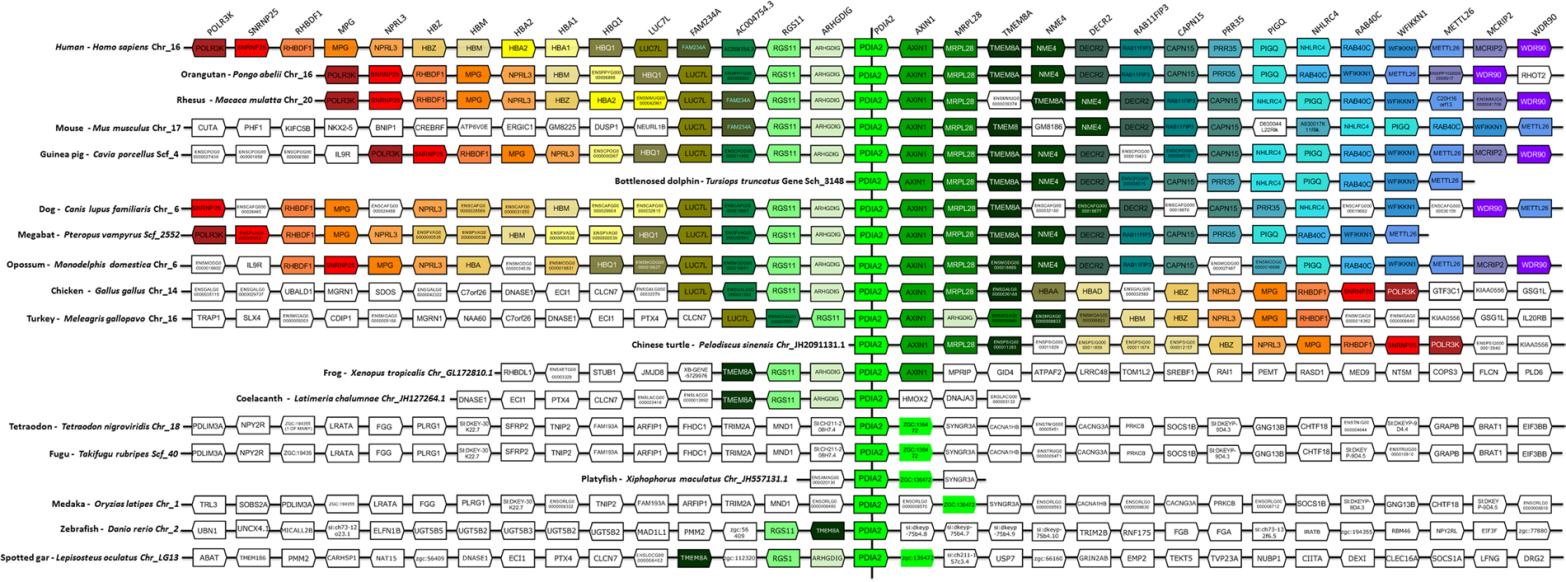
Alignment of the genomic region containing PDIA2 /RhoGDIγ cluster in vertebrates. Gene names and positioning are based upon the Genomicus database (see methods). All species are aligned against *Homo sapiens*. The map is centralized in PDIA2/RhoGDIγ (ARHGDIG) gene pair. Genes are aligned in columms and kept in the order in which they appear in chromosomes (Chr) and scaffolds (Scf), without consideration for distance, while the transcriptional sense is represented by the pentagon tip. All orthologs are drawn with the same color and the lettering on the top refers to the *Homo sapiens* genes. In addition to the main genes of interest, the neighboring genes are included for reference.

**Figure 5:**
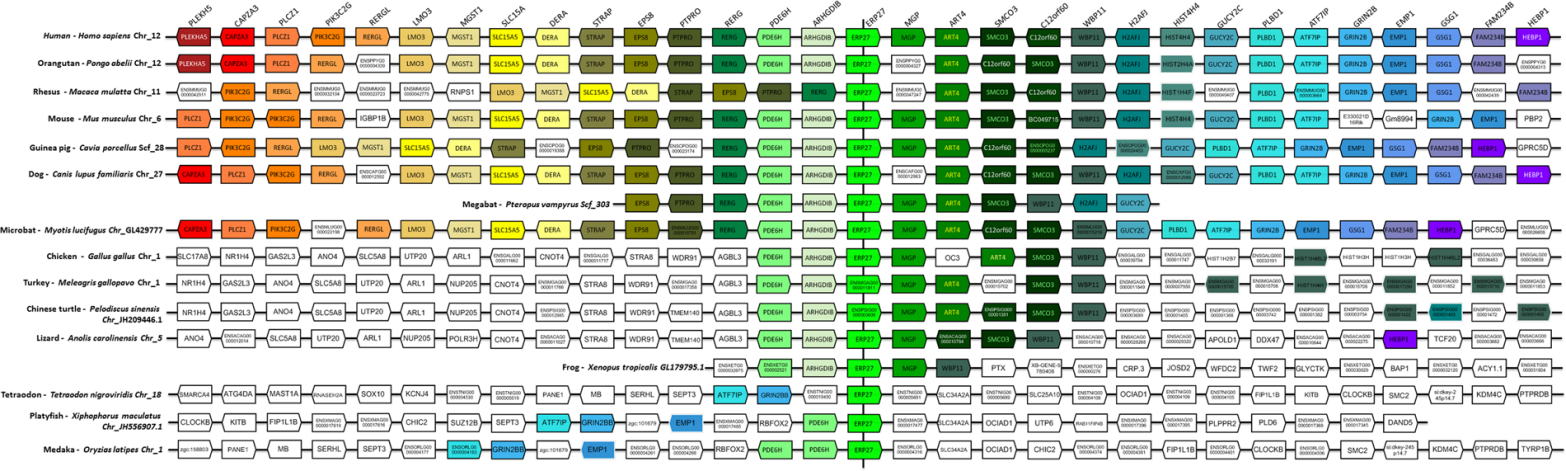
Alignment of the genomic region containing PDIA8/RhoGDIβ cluster in vertebrates. Gene names and positioning are based upon the Genomicus database (see methods). All species are aligned against *Homo sapiens*. The map is centralized in PDIA8 (Erp27)/RhoGDIβ (ARHGDIB) gene pair. Genes are aligned in columms and kept in the order in which they appear in chromosomes (Chr) and scaffolds (Scf), without consideration for distance, while the transcriptional sense is represented by the pentagon tip. All orthologs are drawn with the same color and the lettering on the top refers to the *Homo sapiens* genes. In addition to the main genes of interest, the neighboring genes are included for reference.

We next aimed at dating the evolutionary origin and analyzing the conservation of the three human PDI/RhoGDI gene clusters. We determined the position of orthologs to the genes belonging to the three human PDI/RhoGDI gene clusters in a taxonomically diverse collection of representative animal species (Figs 1 and 2). Importantly, this revealed extensive conservation of microsynteny, i.e., PDIA1/RhoGDIα, PDIA8/RhoGDIβ and PDIA2/RhoGDIγ, in almost all tetrapods, with similar transcription directions (Figs 3–5, Supplementary Fig. S3). In the coelacanth (*L*. *chalumnae*), the closest aquatic relative to tetrapods, two of the three clusters, PDIA1/RhoGDIα and PDIA2/RhoGDIγ (Figs 2–4), with respective intergenic distances of 84.5 and 198.8 kb and the same transcription directions, are also maintained. Regarding the third cluster, we could detect only RhoGDIβ, while PDIA8 is not annotated in the genome (Fig. 5 and Suppl Fig. S3D).

Gene cluster evolution appears more dynamic in the ray-finned fish (Actinopterygii). The spotted gar *(L*. *oculatus)*, representing the early branching Holostei, exhibits the same gene arrangements observed in mammals for PDIA1/RhoGDIα and PDIA2/RhoGDIγ pairs (Figs 1 and 2). Within the teleostei, the PDI/RhoGDI gene clusters have been largely disrupted either by loss of genes or by genomic rearrangements. In the species analyzed by us, pairwise clusterings of PDIA1/RhoGDIα and PDIA2/RhoGDIγ genes are partially (e.g., in tetraodon (*T*. *nigroviridis*), fugu (*T*. *rubripes*), medaka (*O*. *latipes*), platyfish (*X*. *maculatus*)) or entirely (e.g., in zebrafish (*D*. *rerio*) absent (Figs 3–5 and Supplementary Figure 3). Of note, the Holostei do not share the whole genome duplications that have occurred in their sister lineage, the Teleostei. It is probably for this reason that Holostei share many ancestral gene synteny arrangements with humans^22,23^.

The third vertebrate class, the early branching cartilaginous fish (Chondrichthyes), are only represented by a single species, the elephant shark (*C*. *milii*). Notably, we find the same two clusters, PDIA1/RhoGDIα and PDIA2/RhoGDIγ conserved in the shark genome, suggesting that these gene arrangements already existed in the last common ancestor of the vertebrates (Figs 1 and 2).

Integrating the analysis of gene cluster evolution in a phylogenetic tree provides strong evidence that PDI/RhoGDI microsyntenic cluster arrangements in vertebrates arose by two subsequent cluster duplication events in the last common ancestor of vertebrates (Figs 1 and 2). The considerably poor conservation of the PDIA8/RhoGDIβ cluster might be linked to the modified domain architecture of PDIA8 (Fig. 2 and Supplementary Fig. S4). However, Fig. 1 further shows that the arrangement of a primordial RhoGDI next to a PDI gene is substantially older than the vertebrates. It is detected in echinoderms (*P*. *miniata* and *S*. *purpuratus)* nematodes (*C*. *elegans*), and cnidarians (*N*. *vectensis*), in addition to placozoans (*T*. *adhaerens*) (Fig. 1 and Supplementary Table S1). Given that such cluster arrangement cannot be identified in sponges (*A*. *queenslandica*), ctenophores (*M*. *leydyi*) or in the unicellular closest relatives to the animals (*Monosiga brevicollis and Capsaspora owzarsaki*), the cluster emergence can be dated to the last common ancestor the bilateria share with the cnidarians. However, cluster organization appears to have been disrupted in representatives of arthropods *(D*. *melanogaster*, *D*. *pulex*, *A*. *mellifera)*, and Lophotrochozoa (e.g. the molluscs and annelids).

#### Genomic regions flanking PDI/RhoGDI pairs

Analysis of genomic regions flanking the microsyntenic pairs in humans and other species showed that each pair is embedded within a block of genes which are conserved according to each pair, but dissimilar across the distinct pairs. The conservation pattern of these genes is largely similar to those of their respective PDI/GDI pairs (Figs 3–5 and Supplementary Fig. S3). Yet, across each respective cluster, such neighbor genes exhibit no discernible common functions or structural resemblance. An exception is that each of the 3 syntenic pairs shows additional microsynteny with another set of genes coding for protein phosphatase-1 (PP1) regulatory proteins (Figs 3–5 and Supplementary Fig. S3). Genes coding for PPP1R27 (“PP1 regulatory subunit 27”, DYSFIP) or AXIN1 (PPP1R49, a Wnt signaling pathway component) cluster respectively with PDIA1/RhoGDIα and PDIA2/RhoGDIγ pairs, with intergenic distances (vs PDIA1 and PDIA2) of 8.1 and 0.23 kb in humans and the same transcription directions. The pair PDIA8/RhoGDIβ clusters with PP1 regulator WBP11 (PPP1R165, WW domain- binding protein), however with an intergenic distance to PDIA8 of 110.5 kb and 4 interposed genes. In all three cases, the cluster arrangements for these PP1 regulators are also strongly conserved along vertebrates and disrupted in ray-finned fish. (Figs 3–5 and Supplementary Fig. S3).

#### Analysis of cis-regulatory elements (enhancers)

One of the main reasons for the maintenance of microsynteny over evolutionary timescales spanning hundreds of million years is the sharing of cis-regulatory elements for the adjacent genes^17^. We therefore investigated the occurrence of shared cis-regulatory elements using GeneCards software^24^, which prospects potential enhancer blocks from ENCODE, ENSEMBL and FANTOM databases. We were able to find enhancer sequences described to potentially associate with all PDI-RhoGDI pairs described in this work (Table 2). Since these PDI-RhoGDI pairs display common enhancer blocks, they fulfill the initial requirements to be classified as conserved ancestral microsyntenic pairs or CAMPs^17^.

**Table 2:** *In silico* analysis of potential enhancer sequence blocks associated with PDI-RhoGDI gene pairs.

Gene pair	Enhancer block	TSS distance (kb)*	Size	Location
P4HB-ARGHDIA	GH17F081864	−8.3/2.5	8.9	chr17:81864502–81873384
P4HB-ARGHDIA	GH17F081857	0.1/10.8	6.1	chr17:81857580–81863657
PDIA2-ARGHDIG	GH16F000333	54.3/68.7	7.3	chr16:333802–341081
PDIA2-ARGHDIG	GH16F000342	59.7/74.1	1.3	chr16:342161–343428
ERP27-ARGHDIB	GH12F014927	10.1/32.8	2.4	chr12:14927767–14930181
ERP27-ARGHDIB	GH12F014937	0.6/23.2	2.7	chr12:14937141–14939817
ERP27-ARGHDIB	GH12F014945	−16.2/6.4	19.1	chr12:15111199–15111865
ERP27-ARGHDIB	GH12F014975	−36.5/−13.9	0.4	chr12:14975401–14975800

^*^Distance of the enhancer element from the transcription start site (TSS) of the PDI or RhoGDI gene, respectively. Negative values indicate a localization upstream of the TSS. Abbreviations: ARGHDIA, RhoGDIα; ARGHDIB, RhoGDIβ; ARGHDIG, RhoGDIγ; chr, chromosome; PDIA8, Erp27; P4HB, PDIA1; TFB, Transcription binding site; TSS, transcription start site.

### Evidences for PDI/RhoGDI coexpression/coregulation and physical interaction

#### Analysis of coexpression/coregulation of PDIs and RhoGDIs in sequencing datasets

To address whether the shared synteny between PDI and RhoGDI genes associates with functional connections between these two families, we first searched for their coexpression/ coregulation in response to a variety of interventions. Several publicly available sequencing datasets, including cardiovascular system data (see Methods), were analyzed for the presence of histone modifications, transcription factor binding sites and transcripts for the PDIs and RhoGDIs of interest. ENCODE RNA-Seq transcription data of nine different cell lines showed a correlated gene expression if these genes were expressed (Table 3)^25^. The pair PDIA1/RhoGDIα is equally well expressed among all 9 cell lines; PDIA8 and RhoGDIß display correlated expression pattern in HUVEC and Gm12878, and PDIA2 and RhoGDIγ in HUVEC. These expressions mostly correlated with H3K4me3 and H3K27Ac histone modification patterns found in the ENCODE ChIP-Seq data for HUVEC (Table 3)^25^. PDIA2/RhoGDIγ, on the other hand, displayed only H3K27Ac modifications on each promoter, with H3K4me3 present only on RhoGDIγ, probably due to the short promoter region between PDIA2/RhoGDIy. Analysis by ENCODE of 161 transcription factors by ChIP-Seq.^26,27^ supported these observations: PDIA1/RhoGDIα displayed more transcription factor binding sites than PDIA8/RhoGDIß and only some binding sites were found for PDIA2/RhoGDIγ, reflecting ENCODE’s DNase sites on these regions (Table 3)^25^.

**Table 3:** Analysis of transcription, H3K4me3/H3K27Ac histone modifications, DNaseI sites and number of transcription factors according to ENCODE.

Gene	Encode Expression	Encode H3K4me3	Encode H3K27Ac	Encode DnaseI^1^ around TSS	No. of Encode TFBS^2^
PDIA1	around 2000–22591 reads (in all cell lines)	up to 269 reads	up to 195 reads	125	142
RhoDGIα	around 1000–4720 reads (in all cell lines)	up to 156 reads	up to 174 reads	124	108
PDIA2	up to 20 reads (only in HUVEC)	up to 2 reads	up to 3 reads	39	1
RhoGDIy	up to 129 reads (only in HUVEC)	up to 19 reads	up to 3 reads	45	23
PDIA8	up to 18 reads (only in HUVEC, Gm12878)	up to 6 reads	up to 11 reads	1	5
RhoGDIß	up to 8919 (only in HUVEC, Gm12878)	up to 281 reads	up to 279 reads	54	62

^1^DnaseI sites quantification around transcriptional start site. ^2^Transcription-factor binding sites (TFBS) (+/- 2.8kb of transcriptional start site).

Given the plethora of transcription factor binding sites detected in the promoter region of these genes, it is hard to predict the conditions under which such genes will be expressed (Table 4): PDIs and RhoGDIs were not detectably inducible by Vitamin D in human monocytes (GSE69303)^28^, no differential expression was observed in human blood- versus lymphatic-specific dermal microvascular endothelial cells (GSE74332)^29^ and only minor RhoGDI downregulations occurred upon fluid shear stress in HUVECs (GSE71164)^30^. Also, RNA-Seq data showed that gene pairs were not consistently induced in activated mouse T-cells (+/−DMXAA) (GSE89361)^31^ or Jurkat cells stimulated with PMA and ionomycin (GSE85201)^32^. In HUVEC submitted to hypoxia, there was a complex pattern (Table 4) (GSE70330)^33^. Some interim conclusions about those analyses are that: (**a**) PDIs and RhoGDIs, in line with their housekeeping roles, are substantially resilient to transcriptional activation; b) within these databases, the occasional (often minor) individual changes in gene transcription profile yielded no consistent pattern of coregulation, indicating that the existence of microsynteny does not imply an obligatory universal coregulation among the gene pairs of interest.

**Table 4:** Analysis of transcription, inducibility of the gene pairs and binding of transcription factors to the genes indicated.

Gene	GSE69303	GSE74332	GSE71164	GSE89361	GSE85201	GSE70330	GSE34500	GSE54968	GSE81474	GSE76490
PDIA1	0	0	—	0	0	++	++	++	++	0
RhoDGIα	0	0	—	0	0	—	++	++	++	0
PDIA2	0	0	—	0	0	0	0	++	0	++
RhoGDIy	0	0	—	0	0	0	0	++	0	++
PDIA8	0	0	—	0	0	0	0	++^1^	++	0
RhoGDIß	0	0	—	++	0	0	0	++^1^	++	++

0 = no expression difference; −− = decreased; ++ = induced;1with an intermediate peak at day8; GSE69303, Vitamin D; GSE74332, blood- versus lymphatic-HMVECs; GSE71164, Fluid shear stress; GSE85201, Jurkat PMA/Ionomycin; GSE70330, Hypoxia; GSE34500, p65/Pol II TNFa; GSE54968, EC differentiation; GSE81474, CEC differentiation; GSE76490, iPS to neural differentiation.

Other databases, however, showed evidence for coordinated PDI/RhoGDI expression changes in developmental/inflammatory contexts, as follows (Table 4). First, p65 and Pol II ChIP-Seq data in HUVEC revealed that both PDIA1 and RhoGDIα promoter regions near the transcriptional start sites were bound by p65 and RNA Pol II upon TNF-α stimulation (GSE34500)^34^. Also, in a deep RNA-Seq dataset from Kurian *et al*. (GSE54968)^35^ (Table 4) with stem cells differentiated to endothelial cells, there was co-upregulation of PDIA1/RhoGDIα, PDIA8/RhoGDIβ and PDIA2/RhoGDIγ transcripts in differentiated cells. In human stem cell differentiation to corneal endothelial cells, there was co-upregulation of PDIA1/RhoGDIα transcripts in differentiated cells (GSE81474)^36^, while human pluripotent cells undergoing neural differentiation co-upregulated PDIA2/RhoGDIγ transcripts (GSE76490)^37^; in both cases, the negligible changes in the other pairs possibly reflect cell-type specificity (Table 4). Thus, in specific circumstances related to developmental/inflammatory programs, there is consistency regarding co-regulated expression.

#### Investigation of balanced PDI/RhoGDI protein expression

**Figure 6:**
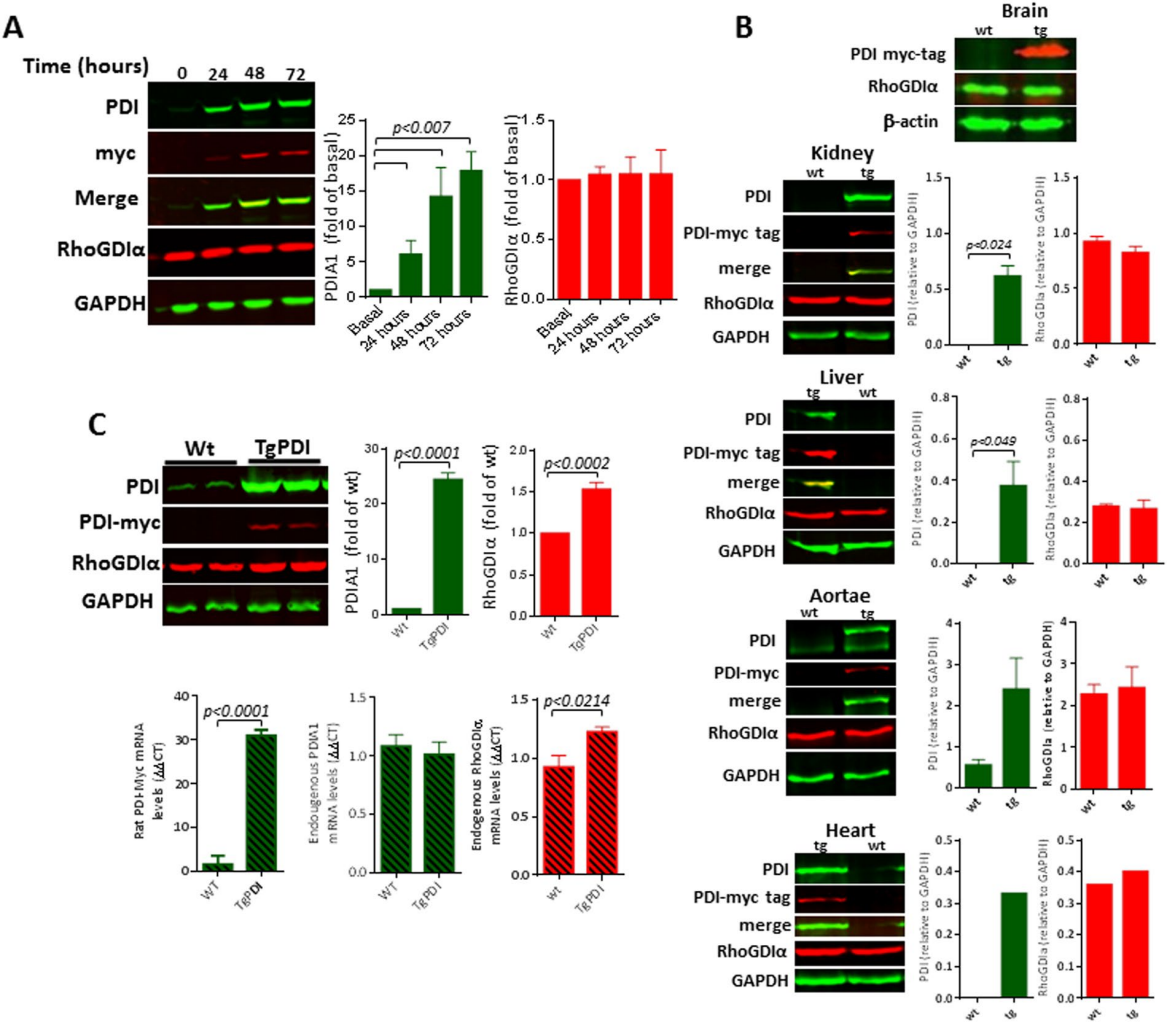
Investigation of PDIA1 and RhoGDIα co-regulation in different systems. (**A)** RhoGDIα protein levels following PDIA1 overexpression in VSMC using a doxycyclin-inducible lentiviral vector carrying a myc-tag; (**B)** Effects of PDIA1 overexpression upon RhoGDIα levels in a transgenic mouse model. Tissue from transgenic PDIA1-overexpressing mice: brain, kidney, liver, aortae and heart. Data representative of n ≥ 3, 2 (heart) or 1 (brain). (**C)** RhoGDIα protein and mRNA levels in VSMC from transgenic PDIA1-overexpressing mice. Representative of 3 independent experiments. Uncropped western blots are shown in Supplementary Figs S8 and S9.

In the next sets of experiments, we investigated the pattern of PDI/RhoGDI coexpression in distinct models, and focused on the PDIA1/RhoGDIα pair, well expressed and relevant in vascular cells. We first investigated whether PDIA1 and RhoGDIα protein expressions are mutually balanced (Fig. 6). For that, we used a lentiviral-carried doxycyclin-inducible system to promote PDIA1 overexpression in VSMC for up to 72 h (Fig. 6A); the increase in PDIA1 expression associates with *ca*. 50% increase in thiol reductase activity by the dieosin reduction assay (unpublished data from our laboratory). Our results indicated no corresponding change in RhoGDIα protein levels. Analogous results were observed also upon acute plasmid-mediated overexpression of PDIA1, PDIA8, RhoGDIγ and PPP1R27 (not shown), as well as down-regulation of PDIA1 or RhoGDIα expression with small-interference RNA in endothelial cells (Supplementary Fig. S5); in each case there were no changes in the expression of their corresponding syntenic partners. In addition, our group recently developed a transgenic mouse model of global constitutive PDIA1 overexpression (unpublished data from our laboratory), which is appropriate to investigate these questions due to its physiological relevance. Distinct organs from transgenic mice, including brain, kidney, liver, aortae and heart showed unaltered RhoGDIα expression (Fig. 6B). These data suggest absence of a mechanism mutually regulating PDIA1/RhoGDIα protein expression balance under unstimulated conditions. In cultured VSMC stimulated with serum, however, presence of the PDIA1 transgene associated with mRNA and protein expression of RhoGDIα (Fig. 6C). In these conditions, endogenous PDIA1 mRNA is unaltered and VSMC exhibit an enhanced proliferative/migratory phenotype (unpublished data from our laboratory).

#### Coexpression of PDIA1 and RhoGDIα upon their promoter activation

**Figure 7:**
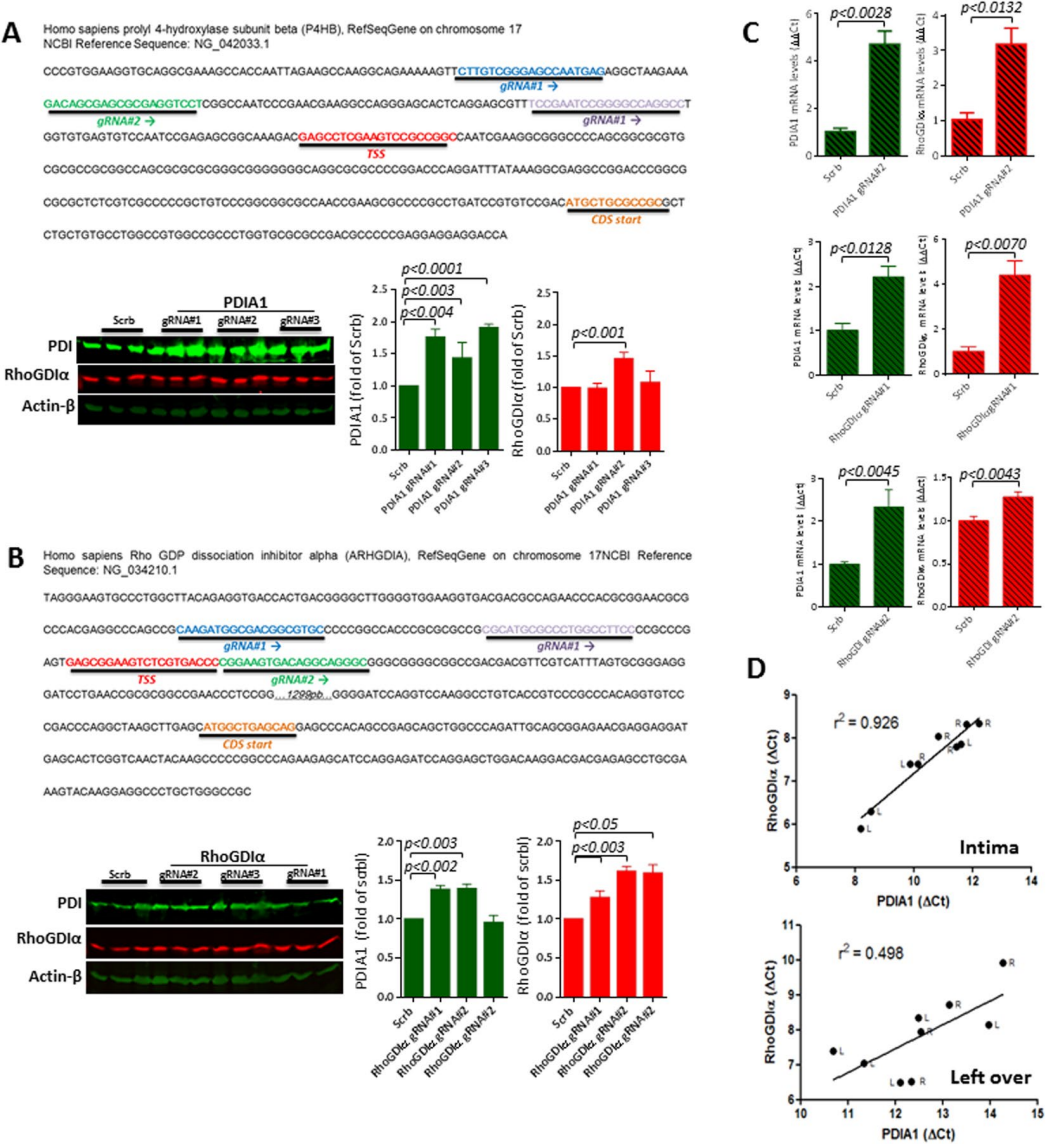
Investigation of PDIA1 and RhoGDIα co-regulation in different systems. (**A)** Effects of induced PDIA1 transcriptional activation on RhoGDIα protein levels (see methods); three distinct guide RNAs, depicted on the map above, were used to drive CRISPR dCas9 VP64-mediated PDIA1 transcription; (**B)** Design similar to (A): effects of induced RhoGDIα transcriptional activation on PDIA1 protein levels; three distinct guideRNAs, depicted on the map above, were used to drive CRISPR dCas9 VP64-mediated RhoGDIα transcription. In both cases, data are representative of three sets of independent experiments. Results are mean and SEM. (**C)** Results of mRNA expression levels from experiments depicted in (**A**) and (**D**). Representative from 3 independent set of experiments; results are mean and SEM. Uncropped western blots are shown in Supplementary Fig. S8. (**D)** Correlation coefficient (r^2^) between mRNA expressions of PDIA1 vs. RhoGDIα from left carotid arteries (**L**) 48 h after partial carotid ligation and their contralateral right carotid arteries (**R**). Left graph depicts intimal samples and right graph depicts left over tissue (media + adventitia). Data represent Delta Ct (gene target - 18 S). Each dot represents an individual artery from n = 4–5 mice.

Manipulation of PDIA1 and RhoGDIα genes by CRISPRa dCas9 VP64 promoter activation system was used to assess PDIA1/RhoGDIα connections. The dCas9 fused to VP64 system can be used to activate silent endogenous genes and reporters^38–40^. In each case, we used three different guide RNAs (gRNA) to target either PDIA1 or RhoGDIα for activation, with their distinct target regions shown in Figs 7A,B. There was increase in RhoGDIα protein (Fig. 7A) and mRNA (Fig. 7C) expression after PDIA1 gene activation using gRNA#2, while PDIA1 up-regulation with gRNA#1 and gRNA#3 did not associate with RhoGDIα expression changes. Conversely, induction of RhoGDIα gene activation by gRNA#1 and gRNA#2 promoted enhanced protein (Fig. 7B) and mRNA (Fig. 7C) PDIA1 expression levels, while RhoGDIα up-regulation with gRNA#3 did not associate with PDIA1 expression change. These data suggest that PDIA1 and RhoGDIα may have transcription cooperation mediated by specific gene promoter regions.

#### Coregulation of PDIA1 and RhoGDIα during flow-induced vascular remodeling  *in vivo*

Given the observed PDIA1/RhoGDIα co-upregulation in developmental/inflammatory conditions (discussed above; Table 4), we addressed if correlated gene expression might be observed during (patho)physiological gene programs of coordinated vascular cell repair. For that, we assessed PDIA1/RhoGDIα gene expression in carotid arteries undergoing flow-induced remodeling^41^. In the left, partially ligated carotid artery, there is a known pattern of disturbed oscillatory proatherogenic flow leading to neointimal thickening and remodeling, while the contralateral right artery undergoes minor expansive compensatory remodeling^41^. Analysis of mRNA expression showed significant correlation between the expressions of PDIA1 vs. RhoGDIα in the intimal layer (Fig. 7D). In contrast, the leftover (media + adventitia), which undergoes a less uniform response, showed poor correlation.

#### Protein interaction between PDIA1 and RhoGDIα

**Figure 8:**
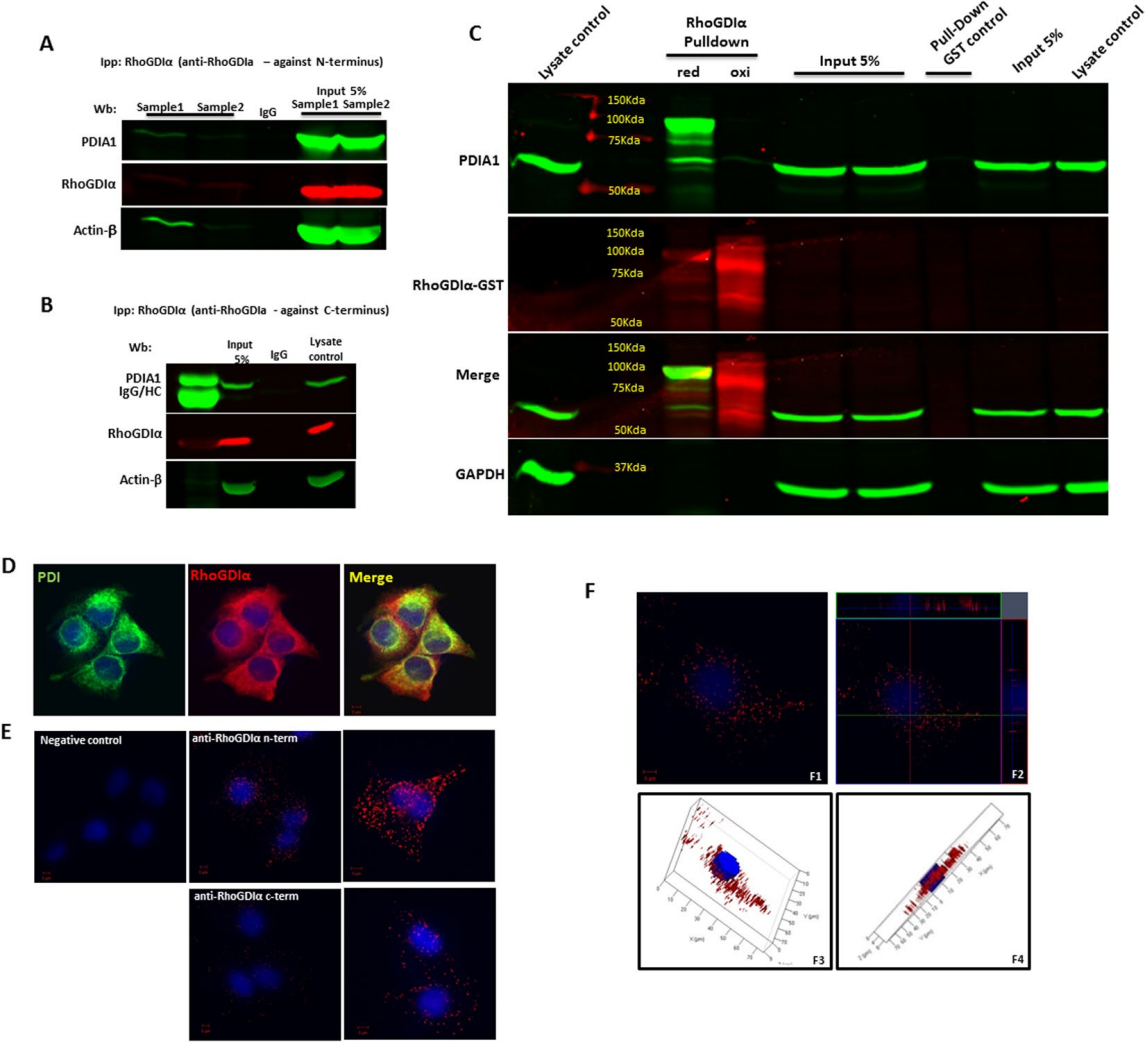
Physical interaction between PDIA1 and RhoGDIα in endothelial cells. (**A**) and (**B)** RhoGDIα immunoprecipitation followed by Western blot for PDIA1. RhoGDIα was immunoprecipitated using IgG against N-terminus (**A**) or C-terminus (**B**). (**C)** PDI and RhoGDIα interaction by pulldown assay. Using RhoGDIα-GST which was either reduced (DTT 20 mM) or oxidized (H2O2 20 mM), we pulled down PDI from HUVEC lysates under reduced conditions. Reduced RhoGDIα was able to pull-down PDIA1 as a monomer and as an apparent heterodimer, while oxidized RhoGDIα incubation resulted in the appearance of its dimeric form. Results representative from 3 experiments. (**D)** Confocal microscopy images for PDIA1 and RhoGDIα showing co-localization. Representative results of three experiments which were replicated using 2 different antibodies to PDIA1 and 2 different antibodies to RhoGDIα (N-terminus and C-terminus). Magnification 40x and zoom 2.5x; Uncropped western blots are shown in Supplementary Fig. S10. (**E)** Representative confocal images of proximity ligation assay (PLA) analysis showing the interaction between PDI and RhoGDIAα in HUVEC. Positive signal of protein interaction is represented as a red dot; nuclei are stained with DAPI (blue). Cells in panels E1-E4 were incubated with a mix of antibodies against PDIA1 and RhoGDIα. Panel B shows negative control in which the primary antibodies were omitted. Magnification: in E, 40x; in F1/F3, 40x and zoom 2x; in F2/F4, 63x and zoom 2x; (**F)** Representative ortho-images (F1 and F2) and 3D reconstruction (F3 and F4) of confocal z-stacks of PLA showing PDI/RhoGDIα dimer distribution into HUVEC. The center image of each panel (shown by crossing lines) is the X-Y view, cross section at the green line is X-Z view and cross section at red line is Y-Z view.

Functional PDIA1/RhoGDIα connections could involve potential interaction between their protein products. PDIA1/RhoGDIα protein interaction was first investigated by co-immunoprecipitation assays in endothelial cell lysates (Fig. 8A,B). Immunoprecipitation of RhoGDIα reproducibly brought a small fraction of PDIA1 (Fig. 8A and Supplementary Fig. S6) using an antibody against its N-terminus, similarly to our previous results in VSMC^37^. To further explore PDIA1/RhoGDIα physical interaction, we performed pull-down assays. Human RhoGDIα–GST fusion protein expressed in a heterologous system was reduced or oxidized, when still immobilized on glutathione-sepharose beads and after purification incubated with endothelial cell lysates (see Methods). RhoGDIα, mainly in the reduced state, was able to pull-down PDIA1, both as a monomer and as an apparent heterodimer (Fig. 8C, merge of bands in yellow ≈100 kDa), and GST control pull-down assays revealed no PDIA1. A RhoGDIα homodimer was also identified (mainly with the oxidized protein pull-down), in line with a previous report showing its homodimerization via hydrophobic interactions^42^. While the redox state of RhoGDIα seemingly influences its interaction with PDIA1, the interaction itself may be independent of redox cysteines, as the PDIA1/RhoGDIα heterodimer was stable under reducing conditions. Confocal microscopy experiments in endothelial cells depicted co-localization (Fig. 8D), with Pearson’s coefficient up to 0.88, confirmed with the use of distinct PDIA1/RhoGDIα antibodies (Suppl Fig. S7). As protein colocalization is limited to allow robust inferences about physical protein interaction, we assessed PDIA1/RhoGDIα physical association by means of proximity ligation assays (PLA Duolink Assay) in endothelial cells, which depicted PDI/RhoGDIα-containing protein complexes appearing as intracellular red dots (Fig. 8E,F), with distinct antibodies. Together, these results provide suggestive evidence that PDIA1 and RhoGDIα display physical interaction in endothelial cells.

## Discussion

Supra-genomic modes of gene regulation associated with conserved chromosomal gene arrangements are increasingly apparent in eukaryotes (reviewed by^17,43^), indicating that placement of genes along eukaryote chromosomes is not random^44–46^, as previously assumed. Broadly speaking, gene positioning may be a relevant mode of genome evolution^18^, and genes are likely arranged in a way to minimize transcriptional noise^47^. However, adjacency at the genomic level is a poor predictor of a functional interaction of genes. Only 4.5% of genes separated by <50 kb are functionally related and only a minority is coexpressed at the protein level^47^. Meanwhile, evolutionarily conserved gene clusters have been proposed to exhibit functional interaction and coregulation^17,18,48–50^, as well studied for beta-globin and Hox genes^51–53^. Such conserved microsyntenic clusters have been increasingly documented in eukaryotes. Accordingly, the conservation of gene order of RhoGDI and PDIA1 genes has been observed before in a study investigating global gene order conservation patterns on a genome-wide scale (Table S2 from)^54^. However, neither the precise evolutionary history nor the functional implications have been further investigated. In fact, a distinctive feature of the evolutionary history of PDI/RhoGDI cluster arrangement in our study is the highly conserved pattern and ancestral origin. The two tandem duplications dating to the LCA of vertebrates, establishing a robust syntenic organization of the three paralog PDI/RhoGDI clusters, apparently coincided with the two rounds of vertebrate genomic duplications^55^. However, a causal correlation between the two events is speculative. Importantly, the structural arrangement between a primordial PDIA and a RhoGDI originated very early in animal evolution, near the LCA that cnidarians share with the bilaterians, i.e., ca. 820 million-years ago. This indicates the presence of a very strong selective pressure to maintain this gene order.

Conserved clustered genes <1 kb apart or genes from the same family in tandem duplication^45,56,57^ were shown to be co-expressed^44,56,57^ or associated with similar metabolic pathways^46,58^, subunits of stable complexes^44,58^ or location at the same subcellular compartment^47^. Along the same line, PDI and RhoGDI genes, despite having unrelated canonical functions, are involved into shared biological processes and, by that, functionally related, as further reflected into the interaction of their protein products. Possibly, such nature of PDI/RhoGDI interaction contributed to prevent gene separation along evolution, since the simple dependence on the same regulatory element, but without any functional correlation, would easily lead, upon duplication of a microsyntenic region, to a reciprocal loss of the duplicated gene. That is, what used to be *Gene1 – Regulator –Gene2* would become upon duplication *Gene1-Regulator-Gene2* and *Gene1′-Regulator*′*-Gene2′*. Subsequent reciprocal loss would lead to *Gene1-Regulator* and *Regulator*′*-Gene2*. However, as the PDI/GDI gene clusters are maintained upon duplication even after > 800 -million years of evolution, this may be cautiously taken as evidence that the genes are not only co-regulated but also functionally linked. As PDIs and RhoGDIs are structurally unrelated housekeeping genes from distinct families, these considerations further highlight functional implications of evolutionarily conserved microsynteny arrangements. A cautionary note, however, is that the general co-regulation and functional interaction among microsyntenic genes has been assumed to a greater extent that in fact demonstrated^19,44,56,57^. While microsynteny conservation could occur essentially by natural selection reflecting functional gene cooperation^45^, in some cases it might alternatively reflect a neutral phenomenon related to low rates of chromosomal rearrangements^59^, although this does not explain the avoidance of reciprocal losses subsequent to duplication.

The major accepted mechanism accounting for microsynteny conservation is the presence of cis-regulatory elements, especially enhancers, that regulate the involved pair, located either within introns between the microsyntenic pair or at a distance, flanking neighbor genes (designated as “bystander genes”), thus constraining the overall genomic block architecture^17,54^. The arrangement of a microsyntenic pair, in which one of the genes is a development-related gene^17^, bystander genes and enhancers characterize the so-called conserved genomic regulatory block^17^. We identified enhancers in the intronic intergenic regions of RhoGDIs or PDIs in our case, together with conserved neighbor genes specific for each syntenic PDI/RhoGDI pair, fittingly with this pattern. Of note, while the typical genomic regulatory block is defined around a trans-dev gene in the microsyntenic pair, PDIs and RhoGDIs are better defined as housekeeping rather than trans-dev genes. Another mechanism potentially supporting the selective conservation of microsyntenic arrangements would be functionally relevant policystronic (conjoined) transcripts from physically contiguous gene pairs^48,60,61^. Evidences for documentation of such transcripts were collected in our survey (see Supp. Table S2 for IDs), including a search through the *ConjoinG* database, which identified a common pre-mRNA transcript covering both PDIA2 and RhoGDIγ. However, the scarcity of these transcripts accross all PDI/RhoGDI pairs and the fact that only the 5′ transcript from the *ConjoinG* database is finally processed^48^ raise doubts about the functional significance of such conjoined transcripts.

Our results significantly extend functional implications for PDI/RhoGDI interactions, further explored here for PDIA1 and RhoGDIα. The resilience of both gene families to expression changes, in response to distinct interventions explored in our datasets, is in line with their housekeeping roles. Such analyses revealed consistency regarding a possible coordinated gene regulation of PDIs and RhoGDIs during stem-cell differentiation and for PDIA1 and RhoGDIα during TNFα responses. Involvement of microsyntenic clusters in developmental processes is a hallmark of the HoxA and beta-globin clusters^54^. Interestingly, we showed evidence for coregulation of PDIA1 and RhoGDIα in the intima of carotid arteries during flow-induced remodeling, a context in which intimal cells likely recapitulate developmental programs related to differentiation, proliferation and migration^62,63^. This extends our previous observations indicating roles of PDIA1 in migration, cytoskeleton and RhoGTPase activation^10^, as well as vascular remodeling and mechanoadaptation^8^. In line with the key roles of RhoGTPases in cytoskeletal organization^64^, RhoGDIα silencing inhibits vascular smooth muscle cell migration^15^. Thus, PDIA1 and RhoGDIα effects concur regarding cytoskeletal architecture. Accordingly, our recent data (Tanaka *et al*., unpublished results) indicate that PDIA1 organizes the localized pattern of RhoA activation. Thus, PDIA1 may be both upstream and downstream RhoGDIα/RhoGTPases, while the precise mechanisms involved in such interactions are yet unclear. In this context, our finding of syntenic PP1 regulator genes together with PDI/RhoGDI is relevant, since the PP1 interactome reportedly correlates with cell polarity^65^. Little is known regarding functional implications of the other syntenic pairs; a SNP within the PDIA2/AXIN1 locus associates with bicuspid aortic valve^66^, and AXIN1 belongs to the Wnt pathway, implicating possible developmental processes.

The evidences for transcriptional co-activation (Fig. 6 and 7), as well as physical PDIA1 and RhoGDIα protein interaction in endothelial cells suggest that both *cis* and *trans* mechanisms mediate the functional connection between the two genes. These data reinforce and significantly extend our previous findings in VSMC^10^. PDIA1/RhoGDIα interaction is not trivial, given their distinct canonical subcellular protein localizations, ER for PDIA1 and cytosol for RhoGDIα. While controversial, there is support for a cytosolic PDI pool^13^, including direct interaction between cytosolic PDIA1 and beta-actin^14^ and reported PDIA1 interactions with the cytosolic protein, soluble guanylyl-cyclase^67^. Interestingly, a large draft map of highly conserved protein complexes across metazoans, consisting of > 1million high-confidence interactions, revealed one large complex of 16 proteins, among which PDIA1 and RhoGDIα are present, also including ezrin, radixin and moesin (known RhoGDI ligands) plus calreticulin, actin-binding and metabolic enzymes^68^. Given our pull-down assays did not support that the PDIA1/RhoGDIα association occurs via redox cysteines (Fig. 8C), plus the lack of dithiol redox motifs in PDIA8, PDI redox motifs in general might not directly mediate their interaction with RhoGDIs. Of note, other previous reports support interaction between different thioredoxin family proteins and GTPases/GDIs: Rho5GTPase/Thioredoxin reductase-1^69^, Rab5/TXNL1 and RabGDI/TXNL1^70^, RhoB/TXNDC3^71^. Also, an Erp57 GDI-like activity regulating RalA signaling has been identified^72^.

Altogether, the remarkably conserved pattern of microsynteny involving PDI and RhoGDI genes, as well as their evolutionary history and functional validations, clearly underscore a relevant connection between PDI and RhoGDI family proteins and implicate the role of RhoGDIα and likely of its related RhoGTPases as candidate mechanisms of PDIA1-mediated cytoskeletal effects and vice-versa. Such a strong cooperation pattern between two highly expressed and functionally relevant families may be crucial for cell homeostasis and can have considerable (patho) physiological implications.

## Methods

### Protein data

Protein sequence data were obtained from UCSC (access: http://genome.ucsc.edu/cgi-bin/hgGateway)^73^ and ENSEMBL Genome Browsers^74^ (access: http://www.ensembl.org/index.html), UNIPROT^75^ (access: http://www.uniprot.org) or Pubmed^76^ Consortium. To assess amino acid similarity between human PDI(s) and RhoGDI(s) proteins, we used EMBOSS Water Pairwise Alignment Algorithm (http://www.ebi.ac.uk/Tools/psa/emboss_water/)^77^.

### Phylogenetic analysis

Phylogenetic analysis was performed using a likelihood framework. Orthologs to the human genes were predicted with HaMStR_OneSeq.^78^ in a collection of 232 species representing the entire tree of life. Orthologous sequences were aligned with MAFFT^79^, and the best fitting substitution model was inferred with ProtTest^80^. Maximum likelihood trees were reconstructed with RAxML v8^81^ using the PROTGAMMALGF model for the PDI alignment, and the PROTGAMMALG for the RhoGDI alignment, respectively. Statistical branch support was assessed with 100 non-parametric bootstrap replicates. Topological differences between the ML tree and the expected branching pattern according to the species phylogeny were tested for significance using the SH test^82^ as implemented into the RAxML package. Pfam domains^83^ were annotated with hmmscan from the HMMER package^84^. The positions of genes orthologous to the human PDIs and RhoGDIs were obtained, when available, from the Genomicus database^85,86^. For species that are not featured in this database, we inferred the position directly from the genome assembly. The following data sources were used:

*Lepisosteus oculatus* - https://www.ensembl.org/Lepisosteus_oculatus/Info/Index

*Callorhynchus milii* - http://esharkgenome.imcb.a-star.edu.sg/blast/;

*Strongylocentrotus purpuratus v4.2* - http://www.echinobase.org/Echinobase/Blasts;

*Nematostella vectensis v1* - http://genome.jgi.doe.gov/pages/blast-query.jsf?db=Nemve1

*Amphimedon queenslandica* - http://metazoa.ensembl.org/Amphimedon_queenslandica/Tools/Blast;

*Monosiga brevicollis v1* - http://genome.jgi.doe.gov/pages/blast-query.jsf?db=Monbr1;

*Capsaspora owzcarzaki* - http://protists.ensembl.org/Capsaspora_owczarzaki_atcc_30864/Info/Index.

### Synteny analyses

Gene synteny and homologs for PDIs and RhoGDIs were initially obtained from the Ensembl database and further analyzed for comparison of the neighboring genomic regions using Genomicus PhyloView of Genomicus Database and Browser^85,86^. Echinoderm genes were identified in EchnoBase Genomic Database^87^. Data were downloaded and characterized manually according the respective data base gene order. All data, gene order, ID numbers and gene distances are depicted in Supplementary Table S3.

### In silico analysis of potential enhancer sequences

For the analysis of potential cis-regulatory elements linked to genome enhancer blocks of PDIs and RhoGDIs of interest to our study, GeneCards software^24^ was applied to prospect potential enhancer blocks from ENCODE, ENSEMBL and FANTOM databases.

### GEO and Encode datasets

The following publicly available datasets were used for analysis: Encode’s 161 Transcription factor binding sites ChIP-Seq (wgEncodeRegTfbsClusteredV3.bed.gz), Encode’s DNase Clustered V3, Encode’s ChIP-Seq data for HUVEC with a-H3K4me1, a-H3K4me3 and a-H3K27Ac, Encode’s Transcription Levels Assayed by RNA-seq on 9 Cell Lines, RNA-Seq of human monocytes stimulated with Vitamin D (GSE69303)^28^, RNA-Seq of human blood- versus lymphatic-specific dermal microvascular endothelial cells (GSE74332)^29^, RNA-Seq of HUVECs subjected to fluid shear stress (GSE71164)^30^, RNA-Seq of activated mouse T-cells (+/−DMXAA) (GSE89361)^31^, RNA-Seq of Jurkat cells stimulated with PMA and Ionomycin (GSE85201)^32^, RNA-Seq of HUVEC under normoxic and hypoxic conditions (GSE70330)^33^, ChIP-Seq of p65 and RNA Pol II upon TNFalpha stimulation in HUVECs (GSE34500)^34^, RNA-Seq of human stem cells differentiated to endothelial cells (GSE54968)^35^, RNA-Seq of human stem cell differentation to corneal endothelial cells (GSE81474)^36^ and RNA-Seq of human inducible pluripotent cells to neural differentation (GSE76490)^37^.

### Ethics

All study protocols were approved by the institutional ethics/scientific committee (Protocol Number 105/12). Experiments with mouse carotid artery ligation were approved by IACUC protocol at Emory University, Atlanta, USA, where they were performed. Experiments with the PDIA1 transgenic mouse model conformed to Guide for the Care and Use of Laboratory Animals (Natl. Acad Sci USA, 1996) and Colégio Brasileiro de Experimentação Animal – COBEA and were approved by the institutional scientific/ethics committee (protocol CEUA 012/12, Ethics Committee of the Clinical Hospital of School of Medicine at University of São Paulo).

### Antibodies

Primary antibodies used for Western blots were as follows: rabbit anti-Myc tag (Cell Signaling, 71D10), mouse anti-β-actin (Sigma Aldrich, A5441), mouse anti-GAPDH (ABCAM, ab8245), rabbit anti-PDI (EnzoLife, SPA890), mouse anti-PDI (Thermo, RL90- MA3019), mouse anti-RhoGDIα (ABCAM, ab135252), mouse anti-RhoGDIα (Santa Cruz, B-10, sc-13120), rabbit anti-RhoGDIα (ABCAM, ab53850), rabbit anti-RhoGDIα (Santa Cruz, A-20 sc360), goat anti-GST (ABCAM, ab6613). Secondary antibodies were fluorescent antibodies from LI-COR.

### Cell culture protocols

Human umbilical vein endothelial cells (HUVEC line, from ATCC) were cultured in RPMI (Gibco) supplemented with 10% FBS, antibiotics and 10 mM HEPES in 95%O2/5%CO2. For experiments, cells (4^th^–8^th^ passage) were trypsinized, pelleted and counted. After that, 2 × 10^6^ cells were collected, centrifuged and homogenized in RIPA buffer (150 mM NaCl, 25 mM Tris-HCl pH 7.4, 1% sodium deoxycholate, 0.1% SDS, 1% NP40, 10 mM MgCl_2_) and phosphatase inhibitors (50 mM sodium fluoride, 2 mM sodium orthovanadate, 10 mM sodium pyrophosphate) or in Trizol for RNA extraction. For VSMC primary culture, VSMC from mice thoracic aortas were isolated by enzymatic digestion from modifications of previously published protocols^88^. Cells were cultured in DMEM (Gibco) low glucose supplemented with 10% fetal bovine serum (FBS, Gibco), 100 mg/ml penicillin and 60 mg/ml streptomycin in 95%O2/5%CO2. For experiments, 1 × 10^6^ cells (4^th^-7^th^ passages) were plated in 100-mm dishes for 4 days. At the 5^th^ day, cells were trypsinized, pelleted and counted. After that, 1 × 10^6^ cells from either wild-type or transgenic mice were collected, centrifuged and homogenized in RIPA buffer supplemented with protease (1 mM PMSF, 1 μg/ml leupeptin and aprotinin) and phosphatase inhibitors (50 mM sodium fluoride, 2 mM sodium orthovanadate, 10 mM sodium pyrophosphate) or in Trizol for RNA extraction.

### Western Blotting

Protein concentration of cell lysates was assessed (BCA method); 30 or 50 μg/ml protein samples were added to sample buffer and electrophoresed in SDS-PAGE system, 12% or 15% gel densities. A semi-dry apparatus (GE HealthCare) was used for transference to nitrocellulose membranes (GE HealthCare). Membranes were incubated in 5% non-fat dry milk for 2 h, washed and incubated with primary antibodies at 4 °C overnight, followed by fluorophore-conjugated secondary antibodies. Bands were detected by Odyssey System (LI-COR Biosciences) and quantified by densitometry using the system program. Uncropped western blots are presented as supplemental material (Supplementary Figs S8–S10).

### Co-immunoprecipitation

HUVEC (1.6 × 10^7^ cells growing in RPMI with 10% FBS) were lysed in lysis buffer (50 mM Tris-HCl pH 7.4, 150 mM NaCl, 1 mM EDTA and 1% Triton × 100) supplemented with protease (1 mM PMSF, 1 ug/ml leupeptin and aprotinin) and phosphatase inhibitors (50 mM sodium fluoride, 2 mM sodium orthovanadate, 10 mM sodium pyrophosphate), plus 1 μM MG132 [proteasome inhibitor] and 10 mM MgCl_2_. Lysates were incubated overnight at 4 °C under agitation with RhoGDIα antibody (rabbit or mouse IgG, Santa Cruz sc-360 (A-20) and sc13120 (B-10), respectively, or rabbit IgG ABCAM ab53850), followed by incubation with 50 μl Protein A-coated magnetic (GE Health Care) beads for 4 h at 4 °C. Beads were successively washed in lysis buffer to remove contaminating material. PDIA1 was detected by western blot using a mouse monoclonal antibody (clone RL90, from Thermo Scientific). Blots were scanned with the Odyssey near-infrared fluorescence imaging system. Results are representative from at least 3 independent experiments.

### RhoGDIα-GST protein expression and pull-down assay

The RhoGDI-GST plasmid was generously donated by Professor Edgar Pick from Sackler School of Medicine, Tel Aviv University. Protein purification was performed according to references^89,90^ with modifications. After the last washing of glutathione sepharose 4B (GE HealthCare), recombinant protein bound to the beads was separated into two aliquots and either oxidized with 20 mM H_2_O_2_ or reduced with 20 mM DTT for 1 hour at 37 °C. Thereafter, they underwent successive washes in 20 mM Tris buffer, pH 8.0. HUVEC were plated and lysates prepared as for the co-immunoprecipitation assays. Cell homogenates were incubated with 200 μg of RhoGDIα-GST (oxidized or reduced) or GST alone, as a control, overnight at 4 °C under agitation. Beads containing recombinant protein bound to captured cell lysate proteins were successively washed in lysis buffer to remove contaminating material. PDIA1 and RhoGDIα-GST were detected by western blot using monoclonal antibodies, respectively antiPDI clone RL90 (Thermo Scientific) and anti-GST (ABCAM).

### Cell transfection

#### (a) Transient plasmid or siRNA transfection

HUVEC plated at 3 × 10^6^ cell density were kept as above and serum-deprived for 4 h, 24 h after plating. Culture medium was replaced by fresh DMEM high glucose medium without serum and antibiotics. Plasmids (20–30 μg) or siRNAs (500–1000 nM) were diluted in transfection medium and lipofectamine according to manufacturer’s instructions (Invitrogen) and incubated with cells for 8 h, followed by change to RPMI plus 10% FBS. Cells were used 24, 48 and 72 h after transfection. b) *Lentiviral-carried Tet-on system:* Inducible PDI overexpression was achieved in rabbit VSMC coinfected with 2 different lentiviruses, one carrying rat PDIA1 gene (with a myc tag inserted at the C-terminus before the C-terminal KDEL sequence, a kind gift of Drs. Tomohiro Nakamura and Stuart Lipton (Burnham Institute for Medical Research, La Jolla, USA) under control of the Tet-inducible promoter/modified Tet-Responsive Element (TREmod), and the regulatory plasmid, which carries the reverse tetracycline trans-activator (rtTA), (Lenti-X Tet-On Advanced Inducible Expression System, Clontech).

### CRISPR dCas9 VP64 promoter activation

Specific guides were designed to target the first 200 bp upstream of the transcription start site (TSS) for both PDIA1 and RhoGDIα (see map at Fig. 6D and E) using a CRISPR designing tool (http://sam.genome-engineering.org/). A scrambled sequence was also designed as control. Sequences were as follows: PDIA1seq. 1-GACAGCGAGCGCGAGGTCCT; PDA1seq. 2-CTTGTCGGGAGCCAATGAG; PDIA1seq. 3-TCCGAATCCGGGGCCAGGCC; RhoGDIαseq. 1-GCCCTGCCTGTCACTTCCG; RhoGDIαseq.2-GCACGCCGTCGCCATCTTG; RhoGDIαseq. 3-GAAGGCCAGGGCGCATGCG; Scrb- GCACTACCAGAGCTAACTCA. Guide oligos were cloned into lenti sgRNA(MS2)_zeo plasmid (a gift from Feng Zhang, Addgene plasmid #61427) according to the Zhang lab SAM cloning protocol available on Addgene (https://www.addgene.org/crispr/zhang/#sam). A nuclease-dead Cas9 construct (lenti dCas-VP64_Blast Addgene plasmid #61425) and a helper complex (lenti MS2-P65-HSF1_Hygro Addgene plasmid #61426) complete this previously described three-vector CRISPR system^91^. For lentivirus production, lenti sgRNA(MS2)_zeo constructs, lenti dCAS-VP64_Blast and lenti MS2-p65-HSF1_hygro were individually transfected into HEK293T cells using lipofectamine 3000 reagent according to the manufacturer’s protocols. psPAX2 (a gift from Didier Trono, Addgene plasmid # 12260), and pCMV-VSV-G (a gift from Bob Weinberg, Addgene plasmid # 8454) were co-transfected with each plasmid above for effective viral particle production; 48 h after transfection, viral supernatants were collected, filtered, and applied to HUVEC after addition of 8 µg/ml of polybrene or frozen at -80 °C for posterior use. We initially transduced HUVEC with dCAS-VP64_Blast and MS2-p65-HSF1_hygro lentiviral particles. After concomitant selection with both antibiotics, we transduced these cells with sgRNA(MS2)_zeo lentiviral particles carrying each of the specific guide sequences and further selected with Zeocyn. The resultant sublines were designated: PDA1 or RhoGDIα gRNA#1, gRNA#2 and gRNA#3.

### Confocal Microscopy

Colocalization experiments were performed as described^8,10^, in HUVEC fixed with 4% PFA and permeabilized with 0.2%NP40, followed by blocking in PBS/BSA 2% for 2 h at 37 °C. Primary antibodies were diluted in PBS/BSA 1% and incubated overnight at 4 °C. Antibodies used were: antiPDIA1 from mouse (Thermo, 1:200) or rabbit (Enzo life, 1:200), antiRhoGDIα from mouse (Santa Cruz, 1:100) or rabbit (Abcam, 1:100). Nuclei were counterstained with DAPI (1:50). Secondary antibodies were Alexa-conjugated, anti-mouse 488 and anti-rabbit 546, both used at 1:400 in PBS/BSA 1% for 2 h at room temperature (from Cell Signaling). For proximity ligation assays (PLA), in the first step we used the same protocol described above for primary antibody incubation. The following steps involved: binding of PLA secondary probed antibodies (PLUS and MINUS), hybridization and ligation of oligonucleotide connectors, final steps for DNA amplification, and labeling with fluorescent probes. These steps were performed according to the Duolink (Sigma Aldrich) protocol manufacturer instructions. Interaction was examined by LSM510 Zeiss Axio Vision laser confocal microscopy (from our local Rede Premium facility).

### Real-time PCR

Real-time PCR was performed as described^8^, using Trizol extraction (Invitrogen). Total RNA was reverse-transcribed using SuperScript II and random primers, and real-time PCR was performed using Platinum SYBR Green qPCR superMix- UDG (Invitrogen) in a StepOnePlus ™ Real-Time PCR System. All reactions were accompanied by a negative control and comparisons were performed by using the Delta cycle threshold (Ct) value (target – housekeeping gene). Results are expressed in fold change (ddCt) and calculated according to the Applied Biosystems guide (http://www6.appliedbiosystems.com/support/tutorials/pdf/quant_pcr.pdf). Primer sequences were as follows: hPDIA1 F:GGCTATCCCACCATCAAGTTC, R:TCACGATGTCATCAGCCTCTC; hRhoGDIα F:TTGACAAGACTGACTACATGGT, R:TGATGGTGAGATTCCACTCCC; hB2M F:CACCCCCACTGAAAAAGATGAG, R:CCTCCATGATGCTGCTTACATG; mPDIA1 F: AAAGGTGGATGCCACAGAAGA, R:GGGTAGCCACGGACACCATA; mRhoGDIα F:AGTTCCTGACACCCATGGAG, R:GCACTTTGGTTTGGGGTAGG; mHPRT F:GCAGCGTTTCTGAGCCATTG, R:AAAGCGGTCTGAGGAGGAAG and rPDImyc F:GGTGAGCGGACACTAGATGG, R:CCTCGGAGATCAGCTTCTGT.

### Mouse models

All experiments with animal models were performed in accordance with the Guide for the Care and Use of Laboratory Animals (Natl. Acad Sci USA, 1996) and Colégio Brasileiro de Experimentação Animal – COBEA. *a) Partial carotid ligation:* Experiments with the mouse model of partial carotid artery ligation were performed with Male C57Bl/6 mice. Briefly, three of four 28 caudal branches of left common carotid artery (L) – left external carotid, internal carotid, and occipital artery - were ligated, while the superior thyroid artery was left intact in isoflurane-anesthetized mice as described. The contralateral right carotid artery (R) was also analyzed, in all cases 48 h after surgery. Total RNA from intima and left over tissue (media + adventitia) was separately obtained from the common carotids at 48 h post-ligation^41^. Briefly, LCA and RCA were quickly flushed with 150 μl of QIAzol lysis reagent (QIAGEN) using 29 G insulin syringe into a microfuge tube. The eluate was then used for intimal RNA isolation according to manufacturer’s instructions (Zymo Research). The RNA from the remaining left over tissue was also extracted after homogenization in QIAzol. Total RNA of each sample was reverse transcribed into cDNA using SuperScriptIII and random primers (Invitrogen). Briefly, qPCR was performed on selected genes using Brilliant II SYBR Green QPCR Master Mix(Stratagene) with custom-designed primers on a Real-Time PCR System (ABI StepOne Plus). Comparisons were performed by using the Delta cycle threshold (Ct) value (target – 18 S). Primer sequences were as follows: PDIA1_Fw:AAGCTGCCGCAAAACTGAAG; PDIA1_Rv:TCACTTCGCTTGAGTCCACC; RhoGDIα_Fw:GACAAGGACGATGAAAGCCTCC; RhoGDIα_Rv:CCTGTCAGGTCCAGTTCCAGAG; 18s_Fw:AGGAATTGACGGAAGGGCACCA; 18s_Rv: GTGCAGCCCCGGACATCTAAG. *b) Transgenic PDI mouse:* Some experiments were performed with a newly-developed model of global transgenic constitutive overexpression of PDIA1 in mice (Fernandes DC *et al*., paper under review). These mice (FVB background) develop and reproduce normally with no gross phenotype. To obtain tissues, animals were euthanized under anesthesia and submitted a perfusion by NaCl 0.9% after laparotomy. The abdominal large vessels were cut to allow release of infused fluid, clean tissues were removed and frozen in liquid nitrogen. After that, 50–100 mg of each tissue was homogenized in RIPA with a Polytron homogeneizer. Thirty to fifty micrograms of protein were used for the western blotting protocol, as describe above.

### Statistical analysis

Statistical analysis was performed using Prism 6 software (GraphPad Software, San Diego, CA). Comparison among multiple groups was performed by one-way analysis of variance, followed by Tukey’s multiple comparison tests. Comparisons between two groups were performed by t-test. Results are described as mean and standard error of the mean (SEM). Linear regression was performed plotting ΔCt for PDIA1 and RhoGDIα (gene target-18S) for goodness of fit (r2) determination of intimal and left over (medial plus adventitial layers). Significance level was 5%.

### Data availability statement

The authors declare that all materials, data and associated protocols are promptly available to readers without undue qualifications in material transfer agreements.

## Electronic supplementary material

Supplementary material

Supplementary Table S-3
