# Supplementary material for: Conserved Gene Microsynteny Unveils Functional Interaction Between Protein Disulfide Isomerase and Rho Guanine-Dissociation Inhibitor Families
Source: Sci Rep. 2017 Dec 8;7:17262. doi: 10.1038/s41598-017-16947-5 (PMC5722932; doi:10.1038/s41598-017-16947-5)
Supplement: Supplementary file 1 — Supplementary material [file 41598_2017_16947_MOESM1_ESM.pdf]

Supplemental Material

Conserved Gene Microsynteny Unveils Functional Interaction Between Protein Disulfide Isomerase and Rho Guanine-Dissociation Inhibitor Families

Ana I. S. Moretti [1#], Jessyca C. Pavanelli [1#], Patrícia Nolasco[1], Matthias S. Leisegang [4], Leonardo Y. Tanaka[1], Carolina G. Fernandes[1], João Wosniak Jr[1], Daniela Kajihara [1], Matheus H. Dias[3], Denise C. Fernandes[1], Hanjoong Jo[2], Ngoc-Vinh Tran[5], Ingo Ebersberger[5,6], Ralf P. Brandes[4], Diego Bonatto[7], Francisco R. M. Laurindo[1*]

# These authors contributed equally to this paper.

From:

[1]Vascular Biology Laboratory, Heart Institute (Incor), University of São Paulo School of Medicine, Sao Paulo, Brazil

[2]The Wallace H. Coulter Department of Biomedical Engineering, Georgia Institute of Technology and Emory University, Atlanta, USA

[3]Special Laboratory for Cell Cycle, Center of Toxins, Immune-Response and Cell Signaling - CeTICS-Cepid, Butantan Institute, São Paulo, Brazil

[4] Institut für Kardiovaskuläre Physiologie, Goethe University, Frankfurt, Germany

[5] Applied Bioinformatics Group, Institute of Cell Biology & Neuroscience, Goethe University, Frankfurt, Germany

[6] Senckenberg Biodiversity and Climate Research Center (BiK-F), Frankfurt, Germany

[7]Department of Molecular Biology and Biotechnology, Federal University of Rio Grande do Sul, Porto Alegre, Brazil

* To whom correspondence should be addressed:

Francisco R M Laurindo

Vascular Biology Laboratory, Heart Institute (InCor), University of São Paulo School of Medicine.

Av. Enéas Carvalho Aguiar, 44, Annex 2, 9th floor, São Paulo, Brazil.

CEP 05403-000; Tel.: +55 11 26615185; FAX: +55 11 2661 5920

E-mail: francisco.laurindo@incor.usp.br

Supplemental Figure Legends

Suppl. Fig. S1 – The maximum likelihood phylogeny of the PDI protein family.
The tree was reconstructed using the LG model of protein sequence evolution, estimating the amino acid equilibrium frequencies from the data. The color code indicates that at least three PDI sub-families that can be traced back to the last eukaryotic common ancestor (LECA), indicated by the filled circles. Open circles denote the position of the last common ancestor of the animals. Black branches represent PDI orthologs that cannot be associated with any of the main PDI lineages. Branch labels denote percent bootstrap support.

Suppl. Fig. S2 – The maximum likelihood phylogeny of the RhoGDI protein family.
The tree was reconstructed using the LG model of protein sequence evolution. The color code indicates the RhoGDI subfamilies that can be traced back to the last common ancestor of the vertebrates (open circles). The filled circle represents the last common ancestor of the animals. Note that the RhoGDIβ lineage (represented in green) has been lost in ray finned fish (actinopterygii) and the shark *C. milii*. Branches in black represent RhoGDI lineages outside the vertebrates. Branch labels denote percent bootstrap support.

Suppl. Fig. S3: Alignment of genomic regions adjacent to RhoGDI genes in selected species in vertebrates.
Gene names and positioning are based upon the Genomicus database (see Methods). All species are aligned against *Homo sapiens*. The map is centralized in RhoGDI genes, focusing on species (particularly ray-finned fish) in which the PDI/RhoGDI arrangement is altered (vs. *Homo sapiens*) in some aspects (gene duplications, displacement of microsyntenic arrangement or absence of the clusters). Data are shown for RhoGDIα (ARHGDIA) (A and B), RhoGDIβ (ARHGDIB) (in C) and RhoGDIγ (ARHGDIG) (in D). Genes are aligned in columms and kept in the order in which they appear in chromosomes (Chr) and scaffolds (Scf), without consideration for distance, while the transcriptional sense is represented by the pentagon tip. All orthologs are drawn with the same color and the lettering on the top refers to the *Homo sapiens* genes. In addition to the RhoGDIs, additional neighboring genes are included for reference.

Suppl. Fig. S4: Identity and similarity (between brackets) of amino acid sequence from human PDIs (A) and RhoGDIs (B) adressed in this study. Proteins are identified by UNIPROT ID.

Suppl. Fig. S5: Effects of PDIA1 or RhoGDIα silencing on the expression of the corresponding pair.
PDIA1 or RhoGDIα were silenced via siRNA transfection and their reduced level (48 h after transfeciton) was unassociated with alterations in their respective partner.

Suppl. Fig. S6: Physical interaction between PDIA1 and RhoGDIα by co-immunoprecipitation.
RhoGDIα were immunoprecipitated with anti-RhoGDI clone A-20 sc360, rabbit IgG. Epitope: N-terminus – against peptide derived of the 25 aminoacids from N-terminus. Western blots: Anti-PDI clone RL90-MA3019, Thermo; anti-RhoGDI clone A-20 sc360, rabbit IgG; anti-actin-B A5441, Sigma Aldrich, mouse IgG.

Suppl. Fig. S7: Physical interaction between PDIA1 and RhoGDIα in endothelial cells.
Confocal microscopic images depicting PDIA1 and RhoGDIα co-localization. In upper and lower panels, antibodies were targeted to the N- and C-terminus of RhoGDIα, respectively. Magnification 40x.

Suppl. Fig. S8: Uncropped western blottings.
A) Western blotting against PDI and RhoGDIα in rabbit VSMC transducing Tet-on system. Representative of 3 samples from three independent experiments (From fig. 6A); **B)** Western blotting against PDI and RhoGDIα in VSMC from PDI transgenic mice. Representative of 9 samples from three independent experiments (From fig. 6C); **C)** Western blotting against PDI and RhoGDIα in HUVEC transducing CRISPR activation system dCas9-VP64 to PDI promoter region (From fig. 7A); **D)** Western blotting against PDI and RhoGDIα in HUVEC transducing CRISPR activation system dCas9-VP64 to RhoGDIα promoter region (From fig. 7B);

Suppl. Fig. S9: Uncropped western blottings
A – E) Effects of PDIA1 overexpression upon RhoGDIα levels in a transgenic mouse model. Tissue from transgenic PDIA1-overexpressing mice: brain (A), kidney (B), liver (C), aortae(D)and heart (E). Data representative of n≥3, 2 (heart) or 1 (brain).,

Suppl. Fig. S10: Uncropped western blottings.

A) RhoGDIα were immunoprecipitated with anti-RhoGDI, ab5385, ABCAM, rabbit IgG. Epitope: N-terminus – against peptide derived of the 25 aminoacids from N-terminus. Western blots: Anti-PDI clone RL90-MA3019, Thermo; anti-RhoGDI ab5385, ABCAM, rabbit IgG; anti-actin-B A5441, Sigma Aldrich, mouse IgG (From Fig. 8A); **B)** RhoGDIα was immunoprecipitated with anti-RhoGDI clone B1- sc-13120, Santa Cruz, mouse IgG. Epitope: C-terminus - aminoacids 166-198.Western blots: Anti-PDI clone RL90-MA3019, Thermo; anti-RhoGDI clone A-20 sc360, rabbit IgG; anti-actin-B A5441, Sigma Aldrich, mouse IgG (From Fig. 8B); **C)** Western blotting against PDI and GST-tag (RhoGDIα) pulldown assay in HUVEC homogenate. Representative of 3 independent experiments (From fig. 8C); **D)** RhoGDIα was immunoprecipitated with anti-RhoGDI clone A-20 sc360, rabbit IgG. Epitope: N-terminus – against peptide derived from the N-terminal 25 aminoacids. Western blots: Anti-PDI clone RL90-MA3019, Thermo; anti-RhoGDI clone A-20 sc360, rabbit IgG; anti-actin-B A5441, Sigma Aldrich, mouse IgG (From Suppl fig. S6);

Supplementary Table S1: PDI/RhoGDI synteny in other organisms.

Protein	Organism	Protein name	Gene ID	mRNA and protein IDs	Genomic sequence	Location	Sense reading	Intergenic distance PDI vs GDI (bases)	Protein Identity/similarity vs human sequences[1]		
									**RhoGDIα**	**RhoGDIβ**	**RhoGDIγ**
**RhoGDI**	*Trichoplax adhaerens*	Putative uncharacterized protein	TRIADDRAFT_53598	XM_002109678.1<br>XP_002109714.1[2]	NW_002060944.1	180,991.....185,870[3]	reverse	6,180	53% / 67%	51% / 69%	47% / 65%
	Trichoplax adhaerens	Putative uncharacterized protein	TRIADDRAFT_20518	XM_002109679.1<br>XP_002109715.1	NW_002060944.1	189,570....193,770	reverse	14,759	52% / 68%	51% / 69%	46% / 63%
	Nematostella vectensis	Predicted Protein	NEMVEDRAFT_v1g170702	XM_001628841.1<br>XP_001628891.1	NW_0018342601	150,219...157,089	sense	7,246	47% / 64%	50% / 67%	40% / 62%
	Strongylocentrotus purpuratus	Sp-Rhogdi/Rho GDP dissociation inhibitor	SPU_025771	SPU_025771.1	------	Scaffold569:<br>461576...467249	reverse	4,909	52% / 67%	49% / 67%	46% / 65%
	Patiria miniata	Sp-Rhogdi_1/Rho GDP dissociation inhibitor	PMI_007749	PMI_007749.1	-----	Scaffold6534:<br>65864...90774	sense	47,978	34% / 44%	34% / 43%	30% / 38%
	Patiria miniata	Sp-Rhogdi_2/Rho GDP dissociation inhibitor	PMI_007751.1	PMI_007751.1	------	Scaffold6534:<br>99795...101554	sense	37,198	49% / 72%	55% / 71%	55% / 70%
									**PDIA1**	**PDIA8**	**PDIA2**
**PDI**	*Trichoplax adhaerens*	Protein Disulfide Isomerase	TRIADDRAFT_52862	XM_00210977.1<br>XP_002109713.1	NW_002060944.1	169,664...174,811	reverse	-------	49% / 69%	24% / 47%	37% / 59%
	Nematostella vectensis	Protein Disulfide Isomerase	NEMVEDRAFT_v1g118540	XM_001628843.1<br>XP_001628893.1	NW_001834260.1	142,973...147,036	sense	-------	58% / 76%	31%/ 51%	42% / 61%
	Strongylocentrotus purpuratus	Sp-Calseq/Protein Disulfide Isomerase [4]	SPU_025772	SPU_05772.1	------	Scaffold569:<br>443358...456667	reverse	-------	46% / 58%	22%/ 41%	35% / 52%
	Patiria miniata	Pm-Calseq/Protein Disulfide Isomerase [5]	PMI_007752	PMI_007752.1	------	Scaffold6534:<br>138752...167074	sense	------	38% / 53%	29%/ 49%	35% / 58%

[1]Uniprot IDs, GDIa, GDIB and GDIg respectivelly, P52562, P52566 and Q99819; and for PDIA1, PDIA8 and PDIA2 are P07237, Q96DN0 and Q13087, respectivelly.
[2]XP_002109714.1 vs XP_002109715.1 = 65% and 77% of identity and similarity, respecitevelly
[3]Intergenic distance between TRIADDRAFT_53598 and TRIADDRAFT_20517 = 3,700 pb.
[4]Sp-Calseq vs human calsequestrins - Casq1 (UNIPROT ID P31415) and Casq2 (UNIPROT ID O14958) : identity/similarity are respectivelly, 21%/37% and 19%/33%
[5]Pm-Calseq vs human calsequestrins - Casq1 (UNIPROT ID P31415) and Casq2 (UNIPROT ID O14958) : identity/similarity are respectivelly, 21%/39% and 21%/39%
T.Adhaerens and N. vectensis source of data: NCBI gene and protein database, UNIPROT.
S. Purpuratus and P. miniata source of data: EchnoBase

Supplementary Table S2: Polycistronic gene transcripts in different organisms

Organism	Name	ID	Source
Homo sapiens	ARHGDIG, PDIP polycistronic mRNA for Rho GDP dissociation inhibitor gamma, pancreatic protein disulfide isomerase, complete cds	AB127078.1	GeneBank
Mus musculus	Rho GDP dissociation inhibitor (GDI) gamma (strand-) contains the whole PDIA2 transcript	ENSMUST00000176961.7	Ensembl
Macaca mulatta	Axin1 or PPP1R49 (strand -) contains exons of PDIA2	ENSMMUT00000000876.3	Ensembl
Callithrix jacchus	PDIA2 contains Axin1 in the intronic region	ENSCJAT00000057730.1	Ensembl
Canis familiaris	ARHGDIB contains exons of ERP27/PDIA8	ENSCAFT00000020458.3	Ensembl
Meleagris gallopavo	PDIA2 (strand -) and part of an exon exhibit sequences of the gene RGS11, regulator of G-protein signaling (strand +)	ENSMGAG00000008956	Ensembl

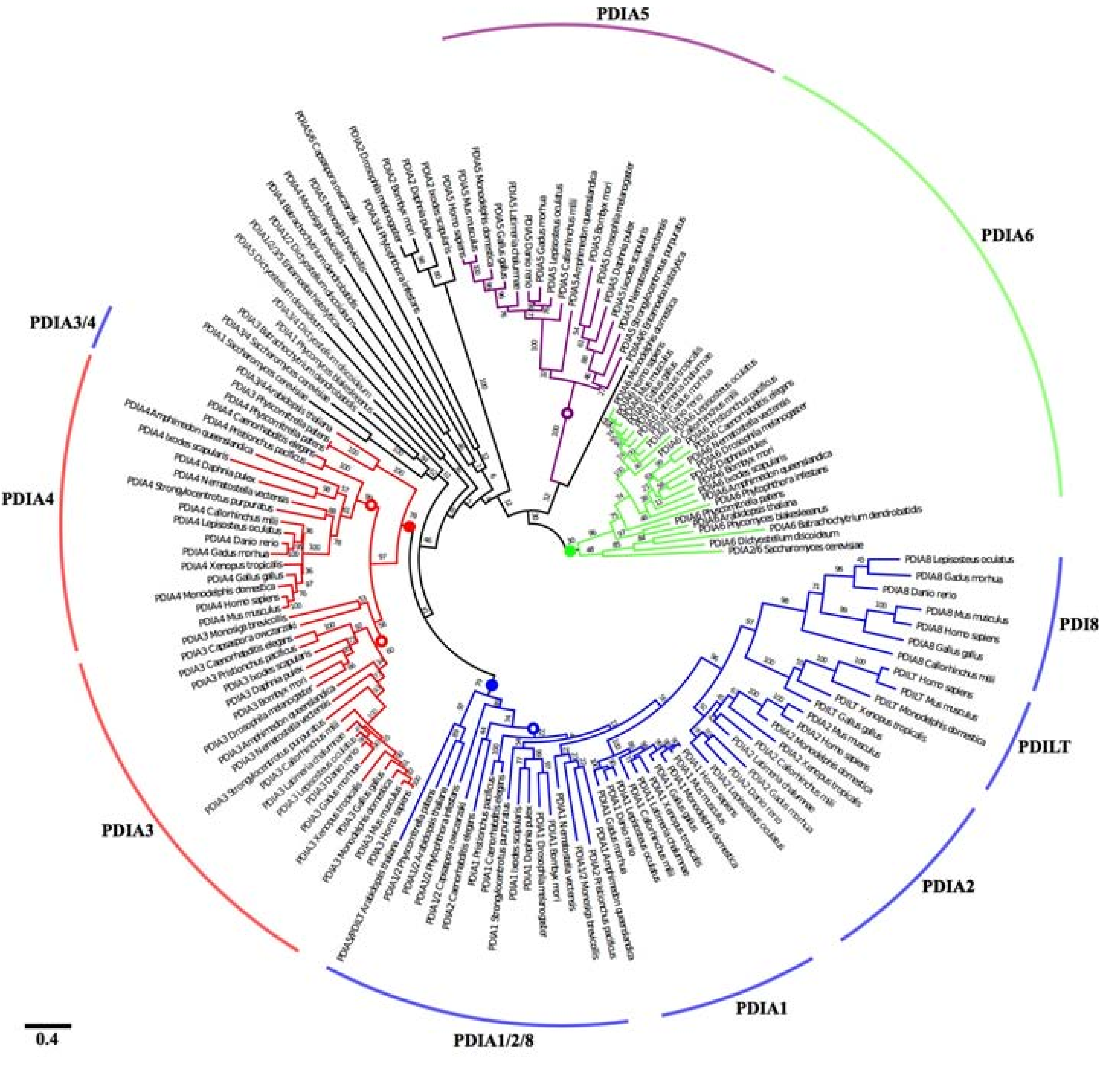
PDIA5
PDIA6
PDIA3/4
PDIA4
PDI8
PDILT
PDIA3
PDIA2
PDIA1
PDIA1/2/8
0.4

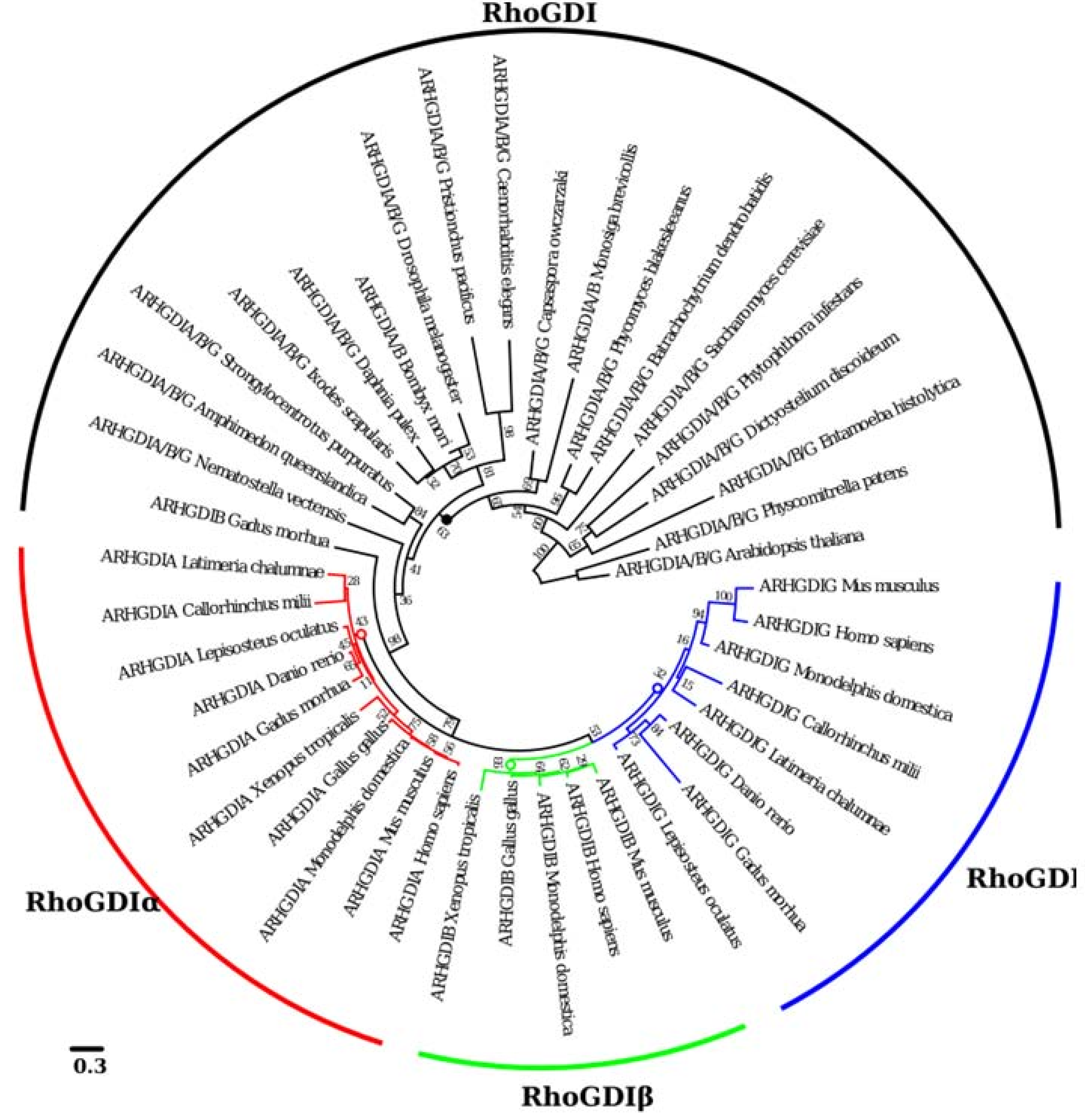
RhoGDI
ARHGDIA/B/G Caenorhabditis elegans
ARHGDIA/B/G Pristionchus pacificus
ARHGDIA/B/G Drosophila melanogaster
ARHGDIA/B Bombyx mori
ARHGDIA/B/G Daphnia pulex
ARHGDIA/B/G Ixodes scapularis
ARHGDIA/B/G Strongylocentrotus purpuratus
ARHGDIA/B/G Amphimedon queenslandica
ARHGDIA/B/G Nematostella vectensis
ARHGDIB Gadus morhua
ARHGDIA/B/G Capsaspora owczarzaki
ARHGDIA/B Monosiga brevicollis
ARHGDIA/B/G Phycomyces blakesleeanus
ARHGDIA/B/G Batrachochytrium dendrobatidis
ARHGDIA/B/G Saccharomyces cerevisiae
ARHGDIA/B/G Phytophthora infestans
ARHGDIA/B/G Dictyostelium discoideum
ARHGDIA/B/G Entamoeba histolytica
ARHGDIA/B/G Physcomitrella patens
ARHGDIA/B/G Arabidopsis thaliana
ARHGDIG Mus musculus
ARHGDIG Homo sapiens
ARHGDIG Monodelphis domestica
ARHGDIG Callorhinchus milii
ARHGDIG Latimeria chalumnae
ARHGDIG Danio rerio
ARHGDIG Gadus morhua
ARHGDIG Lepisosteus oculatus
ARHGDIB Mus musculus
ARHGDIB Homo sapiens
ARHGDIB Monodelphis domestica
ARHGDIB Gallus gallus
ARHGDIB Xenopus tropicalis
ARHGDIA Homo sapiens
ARHGDIA Mus musculus
ARHGDIA Monodelphis domestica
ARHGDIA Gallus gallus
ARHGDIA Xenopus tropicalis
ARHGDIA Gadus morhua
ARHGDIA Danio rerio
ARHGDIA Lepisosteus oculatus
ARHGDIA Callorhinchus milii
ARHGDIA Latimeria chalumnae
RhoGDIα
RhoGDIβ
RhoGDI
0.3

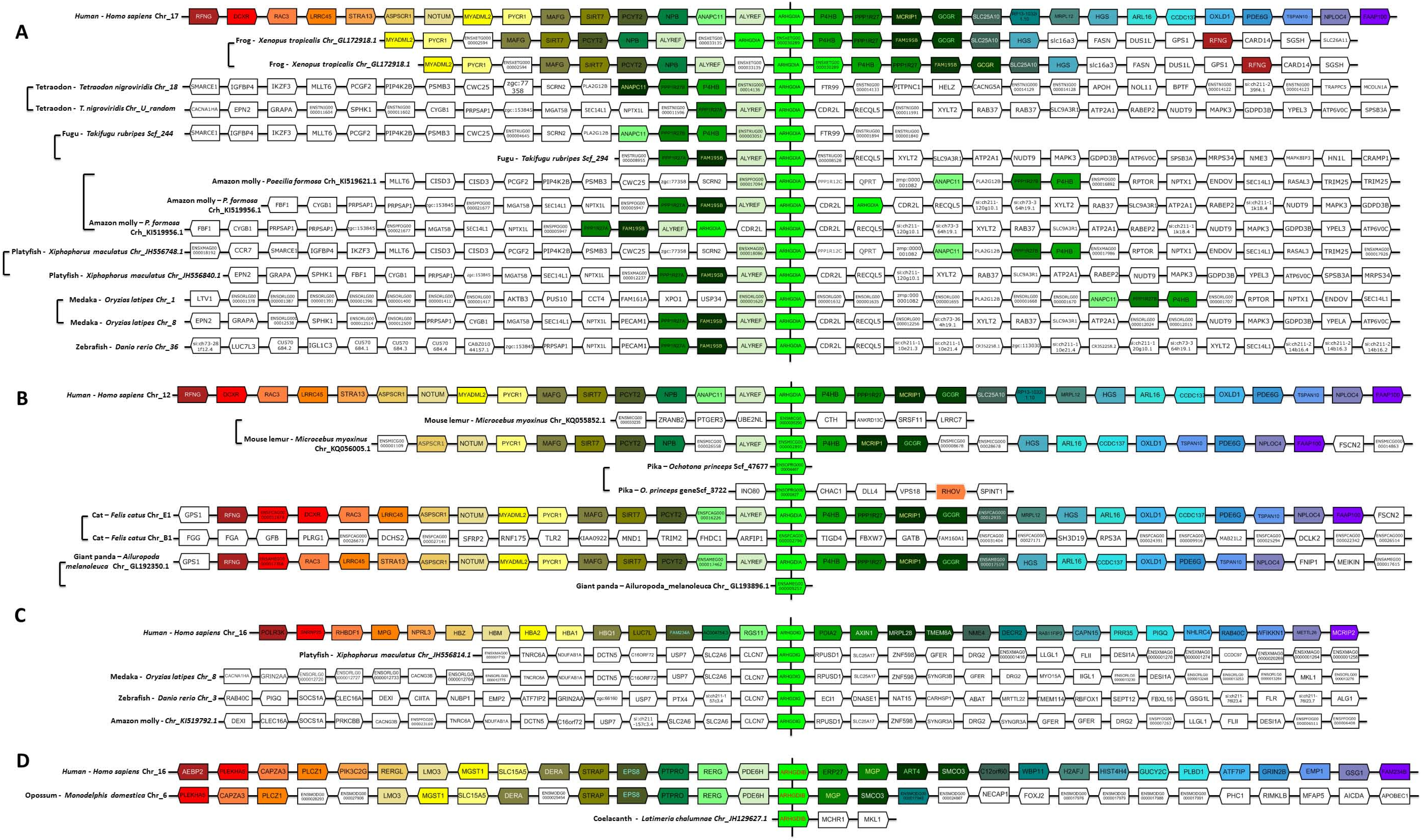

A
Human - Homo sapiens Chr_17
Frog - Xenopus tropicalis Chr_GL172918.1
Frog - Xenopus tropicalis Chr_GL172918.1
Tetraodon - Tetraodon nigroviridis Chr_18
Tetraodon - T. nigroviridis Chr_U_random
Fugu - Takifugu rubripes Scf_244
Fugu - Takifugu rubripes Scf_294
Amazon molly - Poecilia formosa Crh_KI519621.1
Amazon molly – P. formosa Crh_KI519956.1
Amazon molly – P. formosa Crh_KI519956.1
Platyfish - Xiphophorus maculatus Chr_JH556748.1
Platyfish - Xiphophorus maculatus Chr_JH556840.1
Medaka - Oryzias latipes Chr_1
Medaka - Oryzias latipes Chr_8
Zebrafish - Danio rerio Chr_36
B
Human - Homo sapiens Chr_12
Mouse lemur - Microcebus myoxinus Chr_KQ055852.1
Mouse lemur - Microcebus myoxinus Chr_KQ056005.1
Pika – Ochotona princeps Scf_47677
Pika – O. princeps geneScf_3722
Cat – Felis catus Chr_E1
Cat – Felis catus Chr_B1
Giant panda – Ailuropoda melanoleuca Chr_ GL192350.1
Giant panda – Ailuropoda_melanoleuca Chr_ GL193896.1
C
Human - Homo sapiens Chr_16
Platyfish - Xiphophorus maculatus Chr_JH556814.1
Medaka - Oryzias latipes Chr_8
Zebrafish - Danio rerio Chr_3
Amazon molly - Chr_KI519792.1
D
Human - Homo sapiens Chr_16
Opossum - Monodelphis domestica Chr_6
Coelacanth - Latimeria chalumnae Chr_JH129627.1

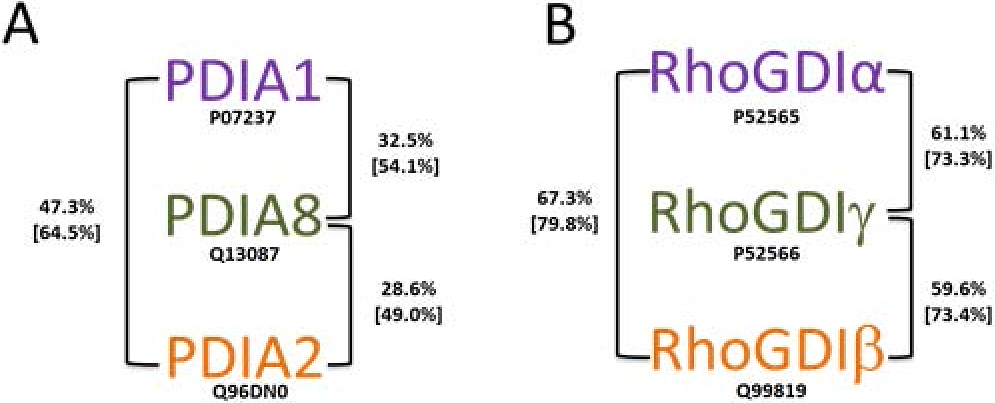

A
PDIA1
P07237
32.5%
[54.1%]
47.3%
[64.5%]
PDIA8
Q13087
28.6%
[49.0%]
PDIA2
Q96DN0
B
RhoGDIα
P52565
61.1%
[73.3%]
67.3%
[79.8%]
RhoGDIγ
P52566
59.6%
[73.4%]
RhoGDIβ
Q99819

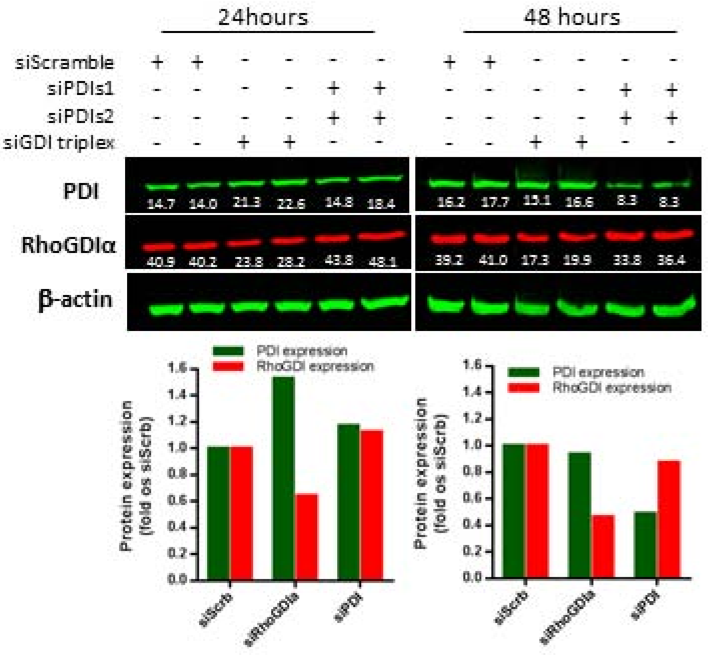

24hours
48 hours
siScramble
siPDIs1
siPDIs2
siGDI triplex
PDI
14.7 14.0 21.3 22.6 14.8 18.4
16.2 17.7 15.1 16.6 8.3 8.3
RhoGDIα
40.9 40.2 23.8 28.2 43.8 48.1
39.2 41.0 17.3 19.9 33.8 36.4
β-actin
PDI expression
RhoGDI expression
Protein expression (fold os siScrb)
siScrb
siRhoGDIa
siPDI

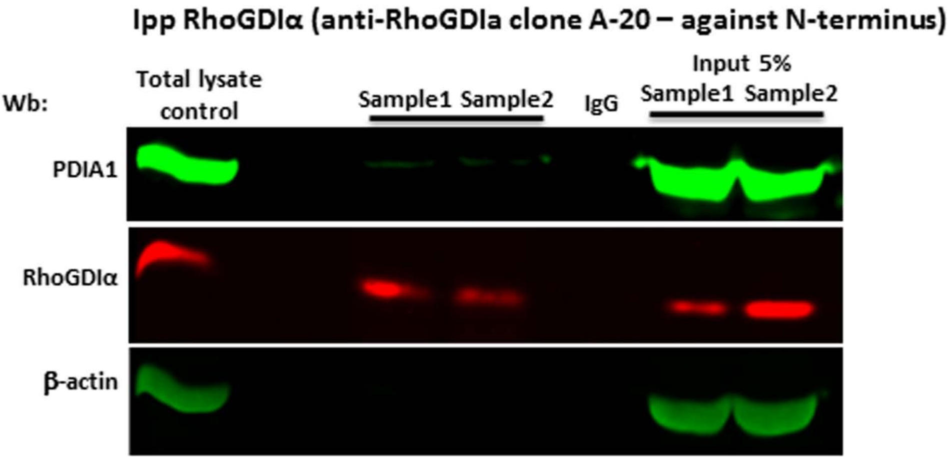
Ipp RhoGDIα (anti-RhoGDIa clone A-20 – against N-terminus)
Wb:
Total lysate control
Sample1 Sample2
IgG
Input 5%
Sample1 Sample2
PDIA1
RhoGDIα
β-actin

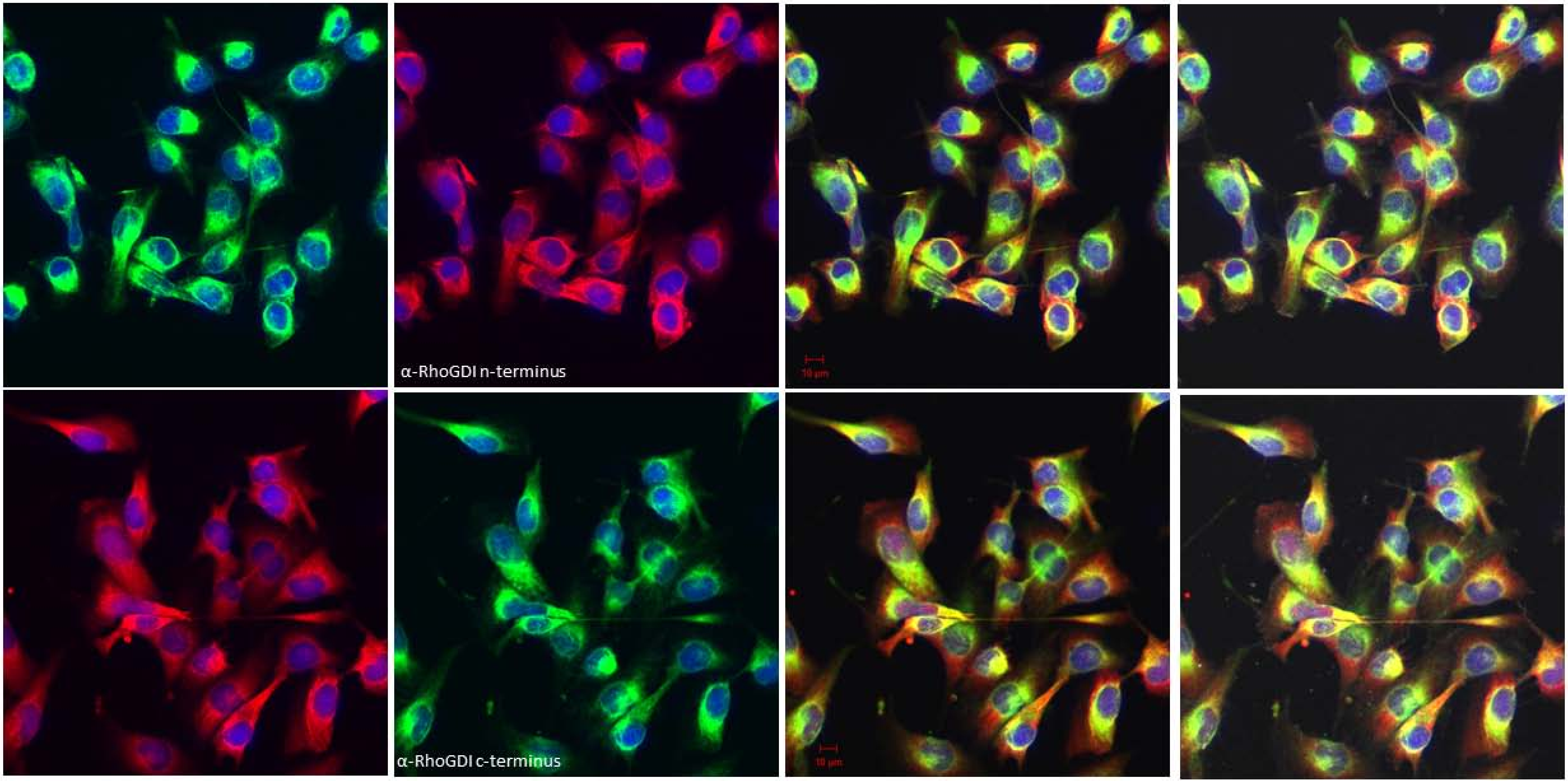
PDI
RhoGDIα
Merge
Phalloidin
α-RhoGDI n-terminus
10 μm
α-RhoGDI c-terminus
10 μm

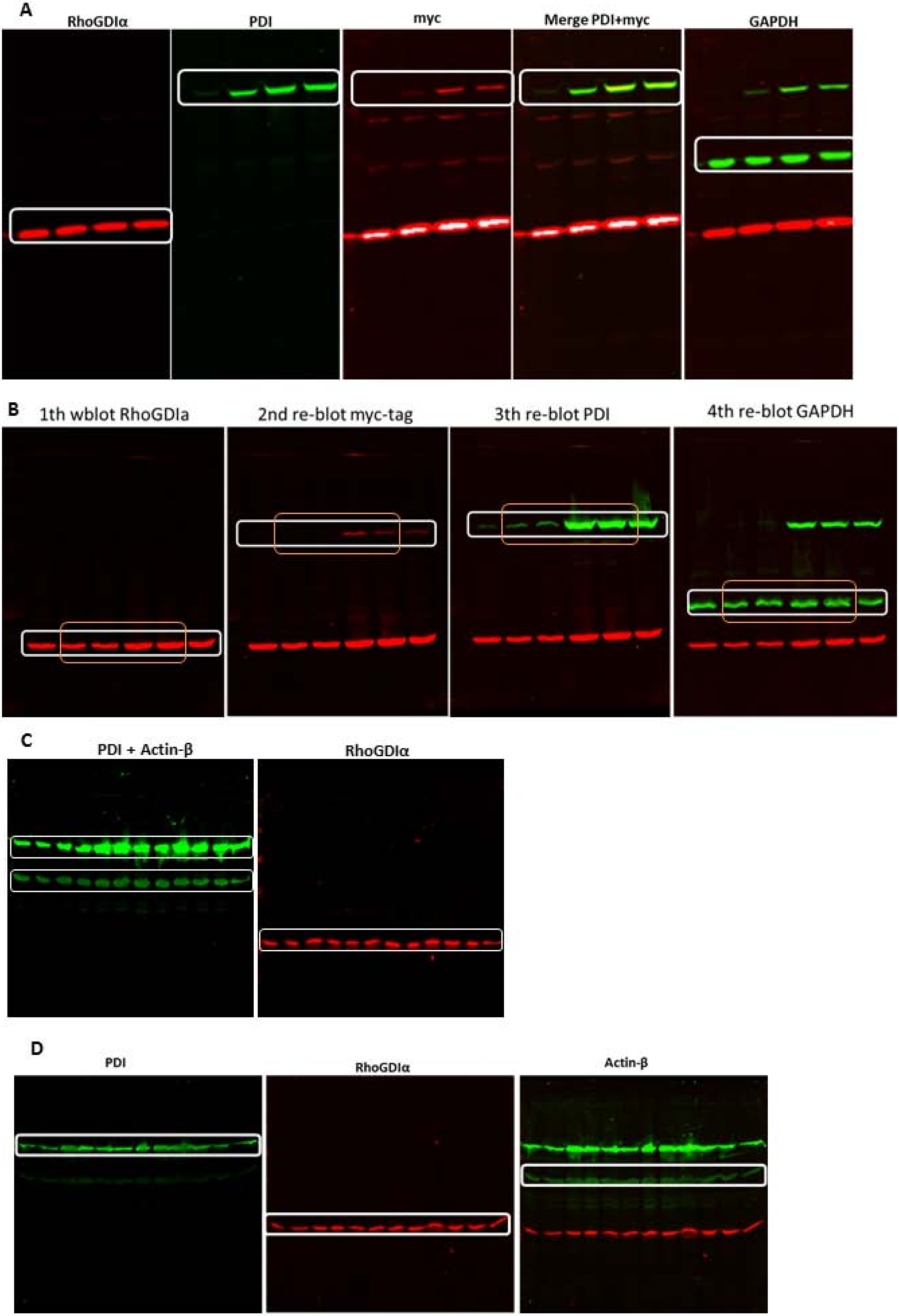
A
RhoGDIα
PDI
myc
Merge PDI+myc
GAPDH
B
1th wblot RhoGDIa
2nd re-blot myc-tag
3th re-blot PDI
4th re-blot GAPDH
C
PDI + Actin-β
RhoGDIα
D
PDI
RhoGDIα
Actin-β

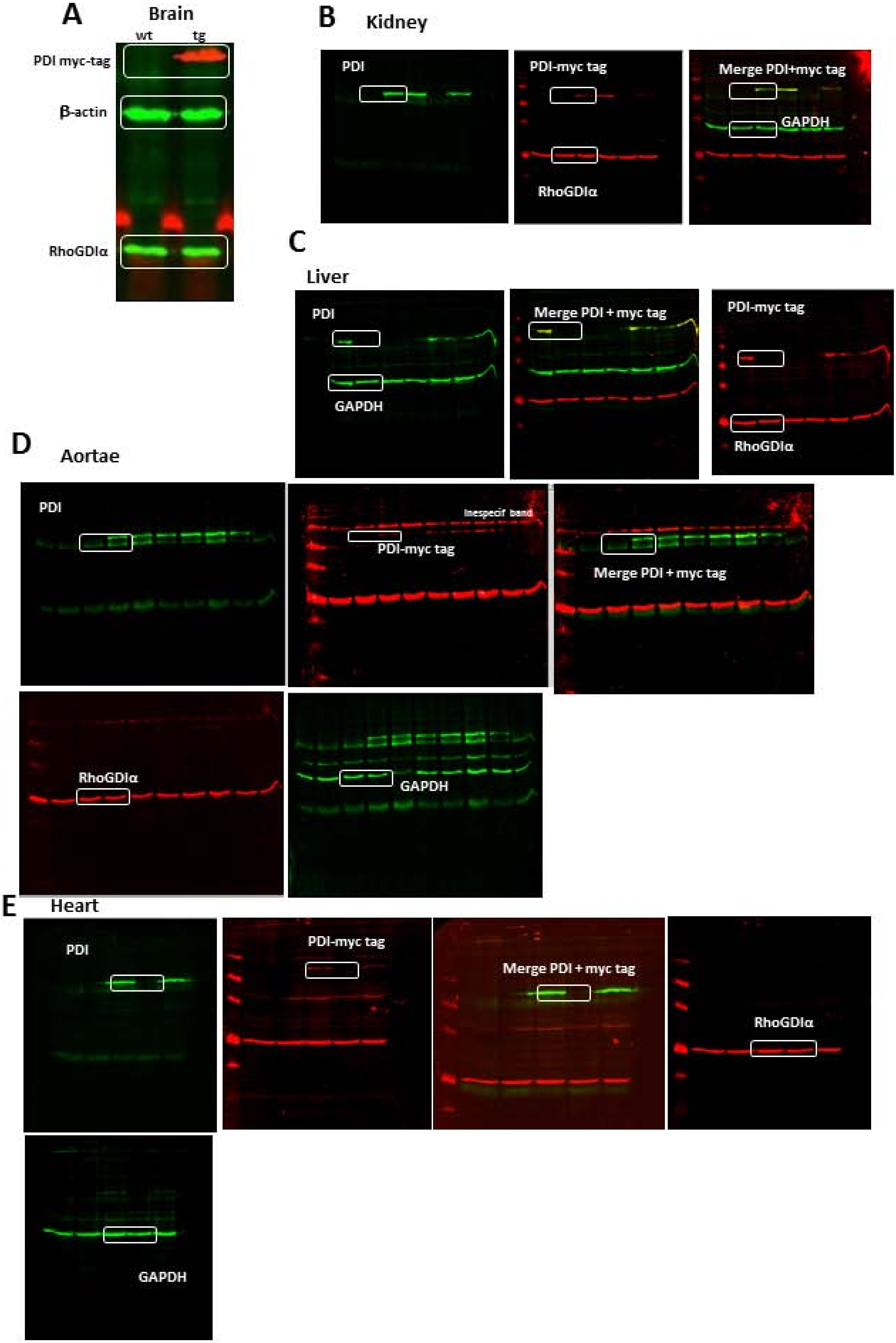

A
Brain
wt
tg
PDI myc-tag
β-actin
RhoGDIα
B
Kidney
PDI
PDI-myc tag
RhoGDIα
Merge PDI+myc tag
GAPDH
C
Liver
PDI
GAPDH
Merge PDI + myc tag
PDI-myc tag
RhoGDIα
D
Aortae
PDI
Inespecif band
PDI-myc tag
Merge PDI + myc tag
RhoGDIα
GAPDH
E
Heart
PDI
PDI-myc tag
Merge PDI + myc tag
RhoGDIα
GAPDH

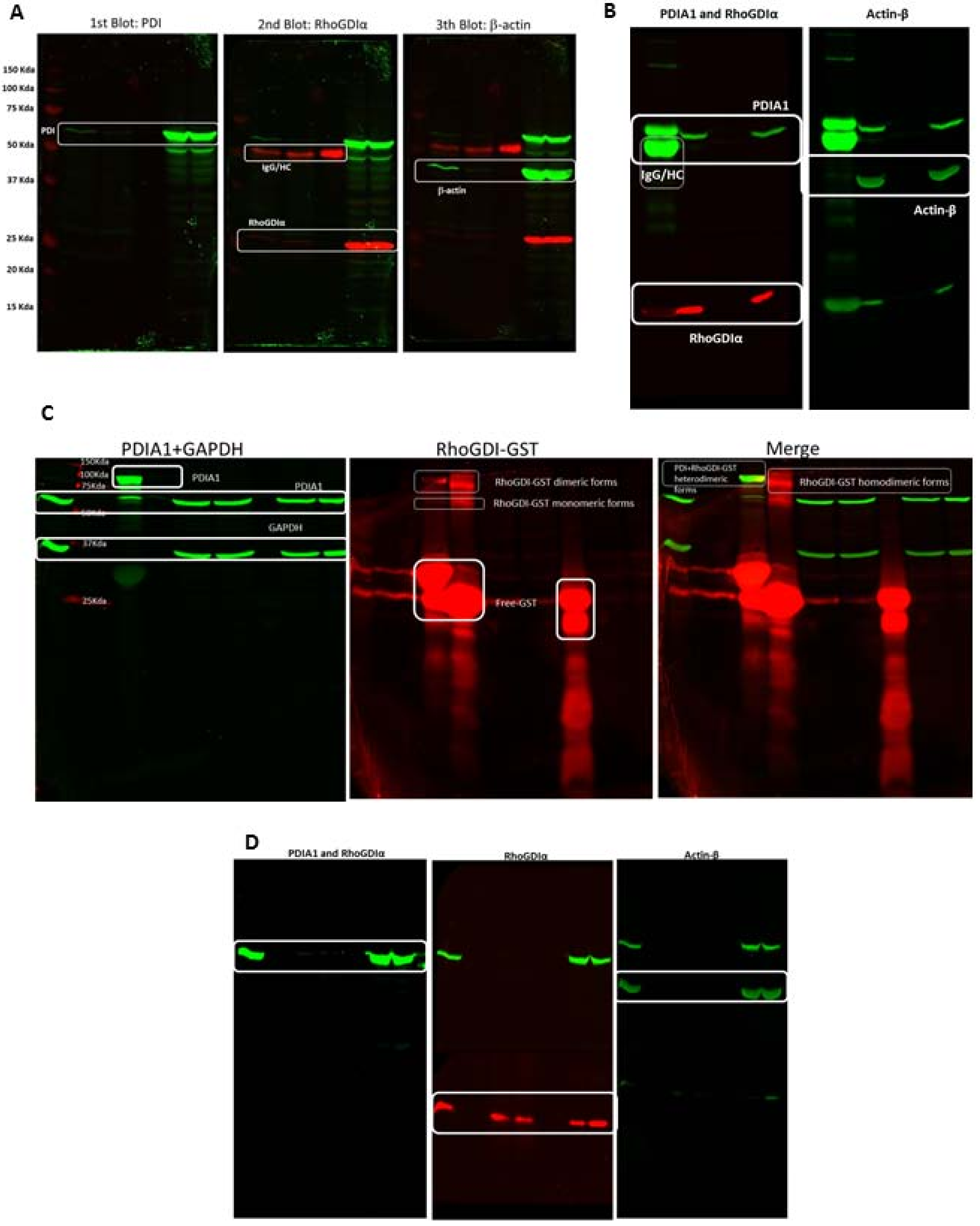
A
1st Blot: PDI
2nd Blot: RhoGDIα
3th Blot: β-actin
150 Kda
100 Kda
75 Kda
50 Kda
37 Kda
25 Kda
20 Kda
15 Kda
PDI
IgG/HC
RhoGDIα
β-actin
B
PDIA1 and RhoGDIα
Actin-β
PDIA1
IgG/HC
Actin-β
RhoGDIα
C
PDIA1+GAPDH
RhoGDI-GST
Merge
150Kda
100Kda
75Kda
PDIA1
PDIA1
GAPDH
37Kda
25Kda
RhoGDI-GST dimeric forms
RhoGDI-GST monomeric forms
Free-GST
PDI+RhoGDI-GST heterodimeric forms
RhoGDI-GST homodimeric forms
D
PDIA1 and RhoGDIα
RhoGDIα
Actin-β
